# ALPK1 controls TIFA/TRAF6-dependent innate immunity against heptose-1,7-bisphosphate of gram-negative bacteria

**DOI:** 10.1371/journal.ppat.1006224

**Published:** 2017-02-21

**Authors:** Milica Milivojevic, Anne-Sophie Dangeard, Christoph Alexander Kasper, Therese Tschon, Mario Emmenlauer, Claudine Pique, Pamela Schnupf, Julie Guignot, Cécile Arrieumerlou

**Affiliations:** 1 INSERM, U1016, Institut Cochin, Paris, France, CNRS, UMR8104, Paris, France, Université Paris Descartes, Sorbonne Paris Cité, France; 2 Biozentrum, University of Basel, Basel, Switzerland; 3 Institut Imagine, UMR_S1163, Paris, France; University of Toronto, CANADA

## Abstract

During infection by invasive bacteria, epithelial cells contribute to innate immunity via the local secretion of inflammatory cytokines. These are directly produced by infected cells or by uninfected bystanders via connexin-dependent cell-cell communication. However, the cellular pathways underlying this process remain largely unknown. Here we perform a genome-wide RNA interference screen and identify TIFA and TRAF6 as central players of *Shigella flexneri* and *Salmonella typhimurium*-induced interleukin-8 expression. We show that threonine 9 and the forkhead-associated domain of TIFA are necessary for the oligomerization of TIFA in both infected and bystander cells. Subsequently, this process triggers TRAF6 oligomerization and NF-κB activation. We demonstrate that TIFA/TRAF6-dependent cytokine expression is induced by the bacterial metabolite heptose-1,7-bisphosphate (HBP). In addition, we identify alpha-kinase 1 (ALPK1) as the critical kinase responsible for TIFA oligomerization and IL-8 expression in response to infection with *S*. *flexneri* and *S*. *typhimurium* but also to *Neisseria meningitidis*. Altogether, these results clearly show that ALPK1 is a master regulator of innate immunity against both invasive and extracellular gram-negative bacteria.

## Author summary

Epithelial cells line internal body cavities of multicellular organisms. They represent the first line of defense against various pathogens including bacteria and viruses. They can sense the presence of invasive pathogens and initiate the recruitment of immune cells to infected tissues via the local secretion of soluble factors, called chemokines. Although this phenomenon is essential for the development of an efficient immune response, the molecular mechanism underlying this process remains largely unknown. Here we demonstrate that the host proteins ALPK1, TIFA and TRAF6 act sequentially to activate the transcription factor NF-κB and regulate the production of chemokines in response to infection by the pathogens *Shigella flexneri*, *Salmonella typhimurium* and *Neisseria meningitidis*. In addition, we show that the production of chemokines is triggered after detection of the bacterial monosaccharide heptose-1,7-bisphosphate, found in gram-negative bacteria. In conclusion, our study uncovers a new molecular mechanism controlling inflammation during infection by gram-negative bacteria and identifies potential targets for treatments aiming at modulating inflammation during infection.

## Introduction

Intestinal epithelial cells (IECs) are not considered to be professional immune cells. However, they play an important role in immuno-surveillance and contribute to the initial phase of inflammation after infection by invasive bacteria or viruses. They can sense the presence of pathogens and orchestrate, together with resident macrophages, the recruitment of immune cells to sites of infection. IECs sense highly conserved pathogen-associated molecular patterns (PAMPs) via pathogen recognition receptors (PRRs) including Toll-like (TLRs) and NOD-like receptors (NLRs). They also detect cellular stress-induced danger-associated molecular patterns (DAMPs) produced during infection. All these sensing mechanisms result in complex signal transduction cascades regulating the expression of proinflammatory genes coding for cytokines, chemokines and antimicrobial peptides.

*Shigella flexneri* is an enteroinvasive bacterium responsible for shigellosis, an acute intestinal inflammation in humans [1]. After ingestion of contaminated food or water, bacteria reach the large intestine and cross the intestinal barrier by transcytosis through M-cells. Once in the submucosal area, they utilize a type III secretion (T3S) apparatus to induce apoptosis in macrophages and invade IECs from their basolateral side. A T3S apparatus is a syringe-like nanodevice enabling the injection of bacterial effector proteins into target cells [2]. Once effectors have translocated into cells, they can subvert the cellular activities of central host factors to favor bacterial internalization. *Shigella* bacteria then escape the internalization vacuole, multiply within the cytoplasm and use actin-based motility to spread from cell-to-cell within the intestinal epithelium. It has been proposed that the main PRR involved in the direct recognition of *S*. *flexneri* is the NLR NOD1 [3]. This receptor recognizes a component of the peptidoglycan called D-glutamyl-meso-diaminopimelic acid that is part of the gram-negative bacterial cell wall [4]. Upon recognition, NOD1 oligomerises and interacts with the receptor-interacting serine/threonine-protein kinase 2 (RIP2) [5]. This protein associates with the transforming growth factor (TGF)-β-activated kinase 1 (TAK1), and the TAK1 binding protein 1 and 2 (TAB1 and 2) complex. This process leads to the phosphorylation, ubiquitination and degradation of the inhibitory κB (IκB), the nuclear translocation of the NF-κB transcription factor and the transcription of pro-inflammatory genes including the gene coding for interleukin-8 (IL-8). TAK1 is also involved in the activation of the MAPKs JNK, p38 and ERK, which are important for the activation of the transcription factor AP1 [6] and histone H3 phosphorylation. In addition, *S*. *flexneri* infection can also be sensed indirectly via the production of DAMPs. For instance, Dupont *et al*. found that the membrane vacuolar remnants produced after vacuolar lysis are detected by host cells and that the signals produced contribute to inflammation [7]. In particular, the accumulation of diacylglycerol around the bacterial entry site and within membrane remnants activates NF-κB via a mechanism dependent on the CARD–BCL10–MALT1 complex and TRAF6 [8]. Interestingly, *S*. *flexneri* possesses a number of tools downregulating the immune response of infected cells. In particular, several type III effectors interfere with the NF-κB and MAPK pathways to reduce IL-8 expression. For instance, OspG reduces the nuclear translocation of NF-κB by preventing IκB ubiquitination and degradation [9]. OspF reduces transcription via its phosphothreonine lyase activity towards p38 and ERK1/2 and its subsequent impact on chromatin remodeling [10].

Although bacteria manipulate the inflammatory response of infected cells, a massive influx of polymorphonuclear cells is observed in tissues infected with *S*. *flexneri* [11]. ATP, released by intestinal epithelial cells after infection by *S*. *flexneri*, contributes to this inflammation [12]. In addition, a previous study by our laboratory showed that innate immunity during *S*. *flexneri* infection is potentiated by a gap junction-mediated mechanism of cell-cell communication between adjacent epithelial cells [13]. We observed NF-κB and MAP kinase activation in uninfected cells located in the proximity of cells containing bacteria and showed that these bystander cells produced large amounts of inflammatory cytokines including IL-8 and tumor necrosis factor alpha (TNFα). IL-8 was also largely produced in bystander cells after infection with *Salmonella typhimurium* and *Listeria monocytogenes* [13, 14], suggesting that potentiation of innate immunity by cell-cell communication is a common host response to different bacterial infections. This phenomenon also occurs during viral infections. First, Patel *et al*. found that recognition of viral double stranded DNA leads to type I interferon expression in bystander cells via a gap junction-mediated mechanism [15]. More recently, it has been shown that anti-viral immunity can spread via the diffusion of cGMP-AMP through gap junctions; cGMP-AMP then binds to the receptor STING localized at the endoplasmic reticulum, which subsequently induces anti-viral gene expression [16].

Although the control of innate immunity has important physiological consequences during bacterial infection, the molecular basis of its regulation remains poorly understood. Here we performed a genome-wide RNAi screen and identified the proteins TIFA and TRAF6 as critical factors for the control of IL-8 expression during *S*. *flexneri* infection. We show that threonine 9 (T9) and the forkhead-associated domain (FHA domain) of TIFA are both important for the oligomerization of TIFA occurring in infected and bystander cells. This process is required for the subsequent oligomerization of TRAF6 and the activation of NF-κB. We demonstrate that TIFA/TRAF6-dependent IL-8 expression is triggered by the bacterial metabolite heptose-1,7-bisphosphate (HBP). In addition, we identify alpha-kinase 1 (ALPK1) as the critical kinase controlling TIFA oligomerization and show that ALPK1 controls innate immunity in response to the invasive bacteria *S*. *flexneri* and *S*. *typhimurium* as well as to the extracellular pathogen *Neisseria meningitidis*.

## Results

### TIFA and TRAF6 are critical players of innate immunity in *S*. *flexneri* infection

**Fig 1:**
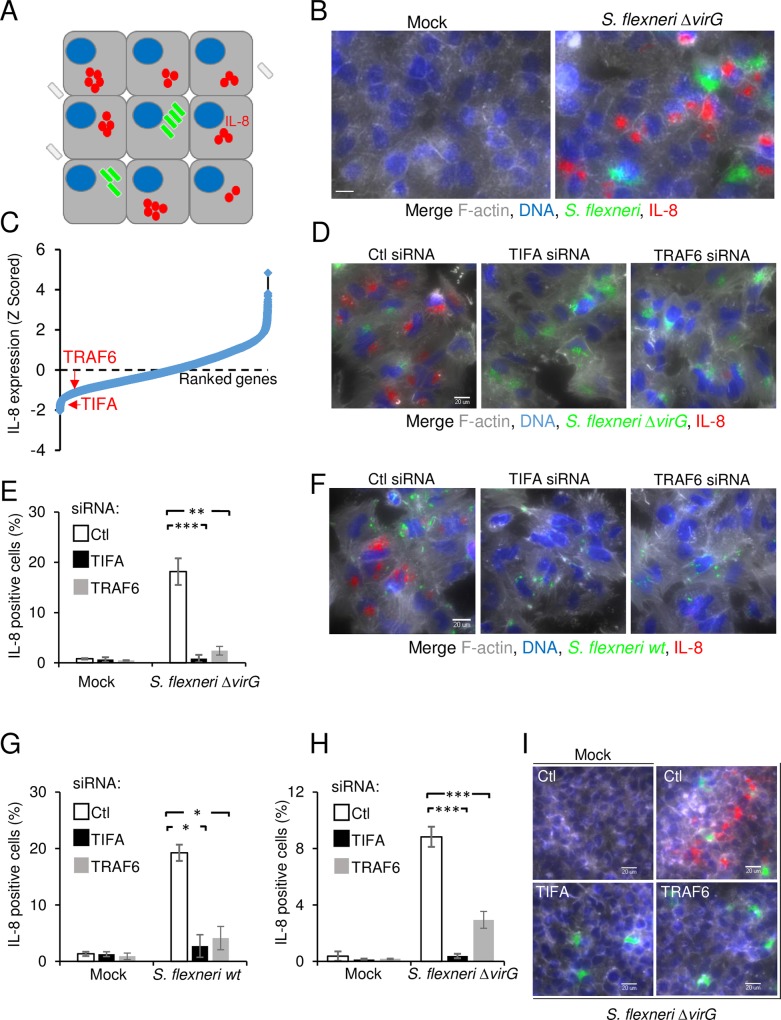
RNAi screen reveals the roles of TIFA and TRAF6 in *S*. *flexneri* infection-induced IL-8 expression. **A**) Schematic representation of the assay used to monitor IL-8 expression in the *S*. *flexneri* infection assay. **B**) Illustration of the image-based assay developed for the screen. HeLa cells were infected for 3.5 hours with *S*. *flexneri* Δ*virG* expressing dsRed under the control of the *uhpT* promoter (green). Cells were stained for F-actin (grey), DNA (blue) and IL-8 (red). Scale bars, 20 μm. **C**) Genome-wide RNAi screening data of IL-8 expression after *S*. *flexneri* infection in HeLa cells. IL-8 measurements were extracted with CellProfiler, Z-scored and ranked. **D**) Validation of the role of TIFA and TRAF6 in *S*. *flexneri* infection-induced IL-8. HeLa cells were transfected with control, TIFA- or TRAF6-targeting siRNAs and infected with *S*. *flexneri* Δ*virG* for 3.5 hours. Cells were stained as in B. **E**) Impact of TIFA and TRAF6 depletion on IL-8 expression. Quantification of cells producing IL-8 as shown in D by automated image analysis (see Methods). Data show the mean +/- SD of 3 independent experiments, p**<0.005, p***<0.0005. **F)** TIFA and TRAF6 control inflammation after wild-type *S*. *flexneri* infection of HeLa cells. Cells were treated as in D and infected with wild-type *S*. *flexneri* for 3.5 hours. **G)** Impact of TIFA and TRAF6 depletion on IL-8 expression. Quantification of cells producing IL-8 as shown in F. Data show the mean +/- SD of 3 independent experiments, p*<0.05. **H)** TIFA and TRAF6 regulate IL-8 expression in HEK293 cells. HEK293 cells were transfected and infected as in D. IL-8 was measured by image analysis. Data correspond to the mean +/- SD of 3 independent experiments, p***<0.0005. **I)** Images showing the implication of TIFA and TRAF6 in HEK293 cells after infection as quantified in H.

In order to characterize the signaling pathways controlling inflammation during infection of epithelial cells by enteroinvasive bacteria, we systematically searched for proteins regulating IL-8 expression following *S*. *flexneri* infection. For this purpose, we developed a high throughput assay that monitors IL-8 expression at the single-cell level using fluorescence microscopy (Fig 1A) and performed a genome-wide RNAi screen. HeLa cells, an epithelial cell line commonly used in *S*. *flexneri* infection assays, were infected for 3.5 hours with the Δ*virG* mutant of *S*. *flexneri* as previously described [17]. This mutant is unable to perform actin-based motility [18] and forms large intracellular microcolonies, which are easily detectable by automated image analysis (Fig 1B and S1 Fig). Background signals from remaining extracellular bacteria were minimized by engineering *S*. *flexneri* to express the dsRed protein only once it is intracellular [19]. dsRed expression was restricted to cytosolic bacteria by placing dsRed under the transcriptional control of the glucose 6-phosphate transporter *uhpt* promoter, which is only upregulated once bacteria are in the presence of glucose 6-phosphate [20]. Cells were then treated with monensin to trap IL-8 in intracellular compartments. After fixation, cells were stained for DNA, F-actin and IL-8 and visualized by immunofluorescence. In agreement with previous work [13], IL-8 expression was largely restricted to uninfected cells located in the proximity of infected cells (Fig 1B and S1 Fig), confirming the importance of bystander cell activation in the control of inflammation during *S*. *flexneri* infection [13, 21]. In order to identify proteins involved in the control of IL-8 expression, the assay was run in a high throughput setup to screen a commercially available genome-wide library made up of pools of 4 siRNAs per gene. Total cell number, infection rates and IL-8 measurements were extracted for all targeted genes using CellProfiler (see Materials and Methods, S1 Table). As expected from previous work [22] [23], pools targeting NF-κB p65 and TAK1 had strong inhibitory effects on IL-8 expression (S1 Table), validating the approach and the experimental setup of the screen. TIFA and TRAF6 were found amongst proteins whose depletion strongly inhibited IL-8 expression after *S*. *flexneri* infection, and were thus selected for further validation and molecular characterization (Fig 1C, S1 Table). TRAF6 mediates signaling from members of the TNF receptor superfamily as well as the Toll/IL-1 family [24]. Interestingly, a previous publication had already reported that TRAF6 was involved in the activation of NF-κB in *S*. *flexneri*-infected cells [8]. TIFA is a 20-kDa protein that was first identified as a TRAF6-interacting protein in a yeast two-hybrid screen [25]. It contains a FHA domain, known to bind phosphothreonines and phosphoserines, and a consensus TRAF6-binding motif [26]. In TNFα signaling, it is involved in the oligomerization of TRAF6, which is required for downstream activation of NF-κB [27]. Very recently, it has been reported that TIFA is involved in the inflammatory response triggered following the detection of heptose-1,7-bisphosphate (HBP), a metabolite present in gram-negative bacteria [28]. HBP can be secreted or released upon bacterial lysis and internalized by eukaryotic cells via endocytosis. In order to exclude possible off-target effects in the RNAi screening data and confirm the specific implication of TIFA and TRAF6 during *S*. *flexneri* infection, silencing of these two genes was repeated with an independent set of siRNA sequences. While infection remained comparable (S2 Fig), this independent approach confirmed a dramatic inhibition of IL-8 after *S*. *flexneri* Δ*virG* infection of cells depleted for TIFA and TRAF6 (Fig 1D and 1E). Similar results were obtained upon infection with wild-type bacteria (Fig 1F and 1G) as well as in HEK293 cells (Fig 1H and 1I), showing that the contribution of TIFA and TRAF6 was not restricted to infections with the Δ*virG* mutant or with HeLa cells. Altogether, these data show that TIFA and TRAF6 play an essential role in the control of inflammation in *S*. *flexneri* infection of epithelial cells and confirm that RNAi screens are valuable tools to identify new players in a given cellular pathway.

### TIFA and TRAF6 control *S*. *flexneri*-induced NF-κB activation

**Fig 2:**
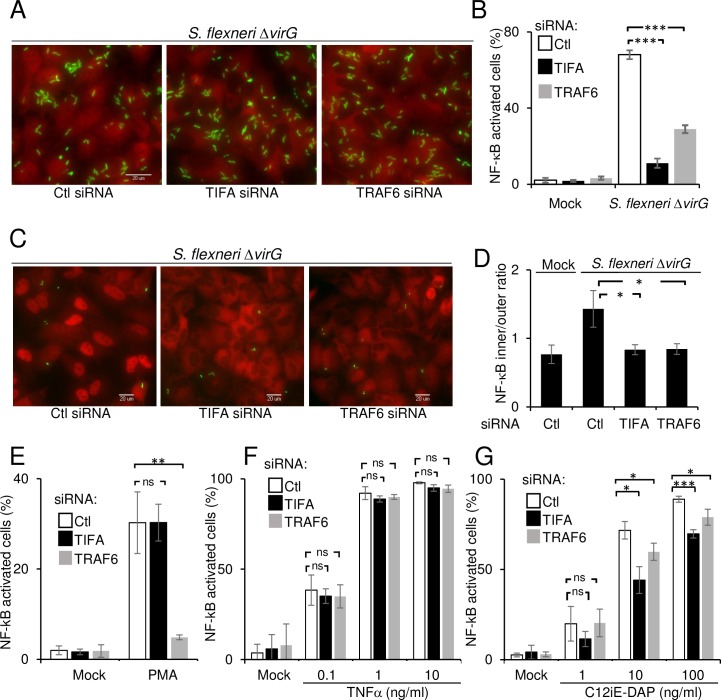
TIFA and TRAF6 control *S*. *flexneri*-induced NF-κB activation. **A)** TIFA and TRAF6 control *S*. *flexneri*-induced NF-κB activation in infected cells. HeLa cells were transfected with control, TIFA- or TRAF6-targeting siRNAs and infected with *S*. *flexneri* Δ*virG* (green) at MOI 20 for 60 minutes. After fixation, cells were stained for NF-κB p65 (red). **B**) Quantification of NF-κB translocation in infected cells after depletion of TIFA and TRAF6. NF-κB translocation was quantified by measuring the intensity ratio between the nucleus and the cytoplasm by automated image analysis, defining a threshold ratio and quantifying the fraction of NF-κB positive cells. Data correspond to the mean +/- SD of triplicate wells from a representative of 3 independent experiments, p***<0.0005. **C**) TIFA and TRAF6 control NF-κB activation both in infected and bystander cells. HeLa cells were treated as in A and infected at a MOI 0.5 (bacteria in green) for 60 minutes. After fixation, cells were stained for NF-κB p65 (red). **D**) Quantification of NF-κB translocation in bystander cells. The fluorescence intensity ratio between the cytoplasm and the nucleus was measured in bystander cells. Data correspond to the mean +/- SD of 3 independent experiments, p*<0.05. **E**) Impact of TIFA and TRAF6 depletion on PMA-induced NF-κB activation. After siRNA transfection, HeLa cells were stimulated with PMA (100 ng/ml) for 60 minutes. Data correspond to the mean +/- SD of 3 independent experiments, p**<0.005, ns: non-significant p>0.05. **F**) Impact of TIFA and TRAF6 depletion on TNFα-induced NF-κB activation. After siRNA transfection, HeLa cells were stimulated for 30 minutes with TNFα at the indicated concentrations. Data correspond to the mean +/- SD of 3 independent experiments, ns: non-significant p>0.05. **G**) Impact of TIFA and TRAF6 depletion on C12-iE-DAP-induced NF-κB activation. After siRNA transfection, HeLa cells were stimulated for 60 minutes with C12-iE-DAP at the indicated concentrations. Data correspond to the mean +/- SD of 3 independent experiments, non-significant p>0.05, p*<0.05, p***<0.0005.

Since a published report indicated that TRAF6 was involved in the activation of NF-κB in *S*. *flexneri*-infected cells [8], we tested whether TIFA was also required for this process. The activation of NF-κB was monitored by following the nuclear translocation of the p65 subunit in conditions where nearly all cells were infected with *S*. *flexneri*. Interestingly, p65 translocation was reduced both in TRAF6 and TIFA-depleted cells (Fig 2A and 2B), showing that these proteins were required to activate NF-κB in infected cells. When cells were infected at a lower MOI (Fig 2C and 2D), a reduction of NF-κB translocation was also found in bystander cells, showing that the depletion of TIFA and TRAF6 had an impact on NF-κB activation in both cell types. The role of TIFA in NF-κB activation was more broadly tested using stimuli other than *S*. *flexneri* infection. In contrast to TRAF6, depletion of TIFA failed to inhibit NF-κB activation induced by phorbol 12-myristate 13-acetate (PMA) (Fig 2E), showing that TIFA is not systematically involved in pathways activating NF-κB and that TRAF6 can also function independently of TIFA. Depleting TIFA and TRAF6 had no significant effect on TNFα-induced NF-κB activation (Fig 2F) but partially inhibited activation induced by the NOD1 ligand C12-iE-DAP (Fig 2G). Together, these results show that TIFA is not involved in the intrinsic machinery of NF-κB activation. Instead, we found TIFA to be implicated in at least two signaling pathways that link bacterial infection to inflammation.

### T9-FHA domain interaction and binding to TRAF6 are required for IL-8 expression

**Fig 3:**
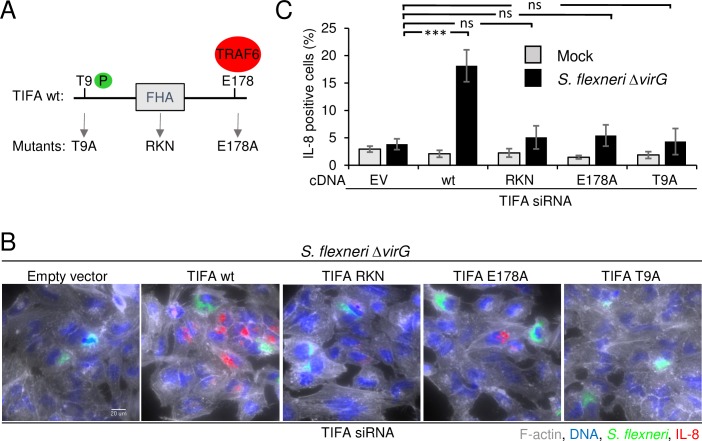
Residue T9, the FHA domain and residue E178 of TIFA are necessary for IL-8 expression. **A)** Schematic representation of wild-type TIFA and the T9, RKN and E178A TIFA mutants. **B**) Only wild-type TIFA rescues IL-8 expression after siRNA-mediated depletion of TIFA. HeLa cells were transfected for 72 hours with TIFA-targeting siRNA. 24 hours prior infection, cells were transfected with empty vector, wild-type or mutated TIFA cDNA constructs. All TIFA cDNA constructs are TIFA siRNA-resistant. Cells were infected with *S*. *flexneri* Δ*virG* (green) for 3.5 hours. After fixation, cells were stained for F-actin (grey), DNA (blue) and IL-8 (red). Scale bars, 20 μm. **C**) Quantification of IL-8 as shown in B. Data correspond to the mean +/- SD of 3 independent experiments, p***<0.0005, ns: non-significant p>0.05.

TIFA contains a FHA domain (Fig 3A), a widespread signaling unit that recognizes phosphorylated threonine and serine residues and binds proteins intra- and inter-molecularly [29]. Huang *et al*. showed that when TIFA is unphosphorylated at the threonine 9 position, it exists as an intrinsic dimer [27]. Upon TNFα stimulation, T9 is phosphorylated by an unknown kinase and FHA-pT9 binding occurs between different dimers forming large TIFA oligomers. This mechanism leads to the subsequent oligomerization of TRAF6 and activation of NF-κB. In order to characterize the mode of action of TIFA during *S*. *flexneri* infection, we investigated the contribution of T9 and the FHA domain. For this purpose, we measured IL-8 expression after infection of cells that were first depleted for TIFA by RNAi and then transfected with siRNA-resistant wild-type or mutated TIFA cDNA constructs. As expected, we found that wild-type TIFA was able to significantly rescue IL-8 expression (Fig 3B and 3C). In contrast, TIFA mutated at T9 (T9A mutant) or within the FHA domain (RKN mutant) failed to restore IL-8 expression. The same result was observed with the TIFA E178A mutant [27], which is unable to bind TRAF6 (Fig 3B and 3C). Altogether, these results show that T9, the FHA domain and E178 are all essential for TIFA activity suggesting that, as in TNFα signaling, the pT9-FHA interaction and the ability to bind TRAF6 are necessary to induce IL-8 expression during *S*. *flexneri* infection.

### TIFA and TRAF6 form co-localizing oligomers in infected and bystander cells

**Fig 4:**
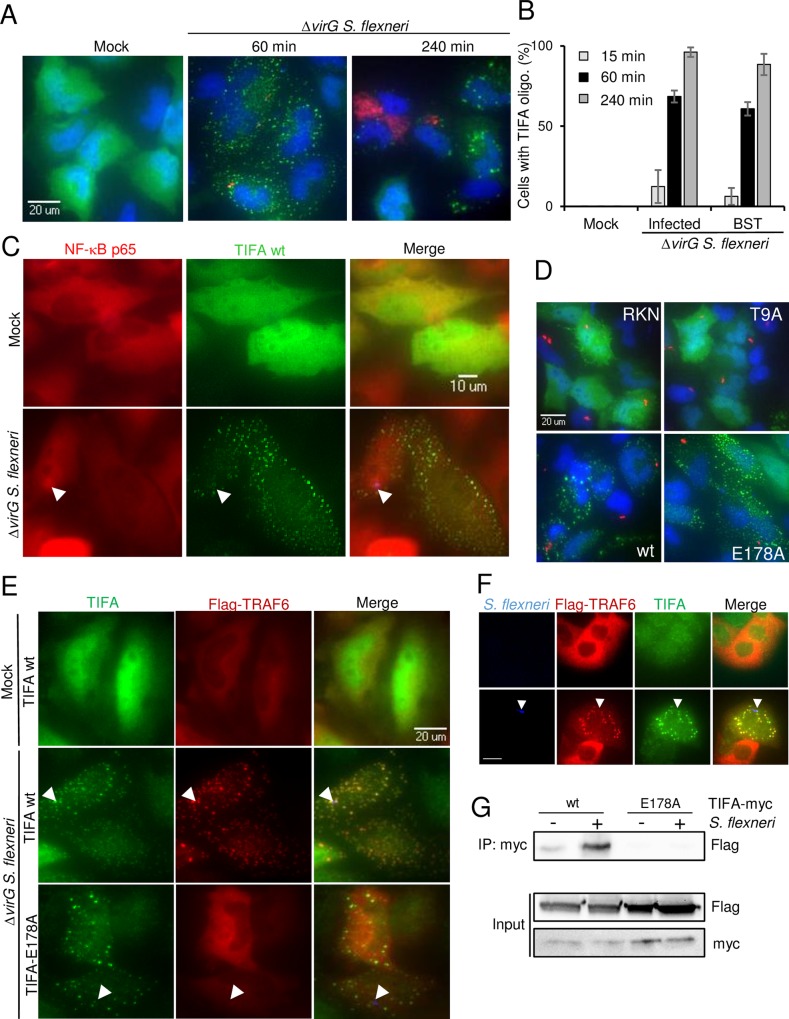
TIFA and TRAF6 form co-localizing oligomers in infected and bystander cells. **A)** TIFA forms large oligomers in infected and bystander cells. HeLa cells were transfected with wild-type TIFA cDNA and infected or not with *S*. *flexneri* Δ*virG* (red) at MOI 0.5. Cells were stained for TIFA (green) and DNA (blue). **B**) Quantification of cells showing TIFA oligomers post infection. Cells were treated as in A. Cells showing TIFA punctuates were manually quantified for infected and bystander cells. Graph shows the mean of triplicate wells with a total of n = 130 cells per condition, data representative of 3 experiments. **C**) TIFA oligomerization occurs within minutes of infection in infected and bystander cells. HeLa cells were transfected with wt TIFA cDNA, infected or not for 15 minutes and co-stained for TIFA (green) and NF-κB p65 (red). Arrows indicate bacteria. **D**) Localization of wt, T9A, RKN and E178A TIFA mutants. Cells were transfected with wt TIFA or the different mutants, infected for 1 hour and stained as in A. Images are representative of three independent experiments. **E**) TRAF6 oligomerization is TIFA-dependent. HeLa cells were co-transfected with wild-type TIFA or E178A TIFA and Flag-TRAF6. After infection, cells were stained for TIFA (green) and Flag (red). Arrows indicate *S*. *flexneri*. **F**) Co-localizing TIFA and TRAF6 oligomers after *S*. *flexneri* infection in Caco-2 cells. Arrows indicate *S*. *flexneri*. Scale bars, 20 μm **G**) Co-immunoprecipitation of TIFA and TRAF6 after *S*. *flexneri* infection. HeLa cells were co-transfected with wt or E178A myc-TIFA and Flag-TRAF6 and infected for 1 hour at MOI 10. Myc IP was blotted with an anti-Flag antibody and the input lysate with anti-Flag and anti-myc antibodies. Data representative of two independent experiments.

In order to better characterize the role of TIFA in *S*. *flexneri* infection of epithelial cells, we monitored its subcellular localization. For this, cells were transfected with a TIFA cDNA construct and TIFA was visualized after infection by immunofluorescence using a TIFA-specific antibody. In the absence of infection, the protein was uniformly distributed in the cytoplasm and the nucleus (Fig 4A). Following infection with *S*. *flexneri*, punctate structures, likely corresponding to large TIFA protein oligomers [27], were formed. These structures were still visible in *S*. *flexneri*-challenged cells after several hours (Fig 4A and 4B). TIFA oligomers were found in both infected and bystander cells, suggesting that TIFA was functionally active in both cell types during infection. A co-staining between TIFA and NF-κB p65 showed that TIFA oligomers formed as early as 15 minutes post-infection and seemed to even precede NF-κB activation as visible in some cells (Fig 4C). TIFA oligomerization was also observed following infection of the Caco-2 cell line (S3 Fig), revealing that this process is also a relevant host response to *S*. *flexneri* infection in human colonic cells.

The role of the FHA-pT9 interaction and TRAF6 binding in the mechanism of TIFA oligomerization was investigated in cells transfected with the different TIFA mutants. Neither the T9A nor the RKN mutant was able to form oligomers (Fig 4D), indicating that the FHA-pT9 interaction was necessary. In contrast, the E178A mutant formed oligomers (Fig 4D), demonstrating that binding to TRAF6 was not required for TIFA oligomerization. Extrapolating these data to the IL-8 rescue experiment (Fig 3B and 3C) suggests that TIFA oligomerization and binding to TRAF6 are both required to induce IL-8 expression after *S*. *flexneri* infection. These results further suggested that, in line with published data on TNFα signaling [27], TIFA also induces the oligomerization of TRAF6 and the subsequent activation of NF-κB following *S*. *flexneri* infection. This hypothesis was tested by determining whether TIFA and TRAF6 co-localized after infection. The localization of both proteins was first visualized in *S*. *flexneri-*infected HeLa cells co-transfected with TIFA-myc and TRAF6-Flag cDNA constructs. As shown in Fig 4E, TRAF6 was also found in punctate structures both in infected and bystander cells. Furthermore, these structures were perfectly co-localized with TIFA oligomers. The same result was obtained upon infection of Caco-2 cells (Fig 4F). Interestingly, the E178A TIFA mutant that is unable to bind TRAF6 did not co-localize with TRAF6 (Fig 4E). The absence of TRAF6 oligomers in these cells showed that the formation of these structures was dependent on the ability of TIFA to bind TRAF6. The interaction between TIFA and TRAF6 was further addressed by co-immunoprecipitation in cells transfected with TIFA-myc and TRAF6-Flag (Fig 4G). A weak signal was detected in uninfected cells showing some TIFA-TRAF6 interaction under basal conditions whereas their interaction was strongly enhanced upon *S*. *flexneri* infection. As expected, this interaction was not observed when cells were transfected with the E178A TIFA mutant (Fig 4G), confirming that TIFA and TRAF6 interact in a TIFA E178-dependent manner. Altogether, these results show that *S*. *flexneri* infection induces the formation of co-localizing TIFA and TRAF6 oligomers and that the TIFA-TRAF6 interaction depends on E178 of TIFA.

### TIFA/TRAF6-dependent innate immunity is triggered by HBP in *S*. *flexneri* and S. *typhimurium* infection

**Fig 5:**
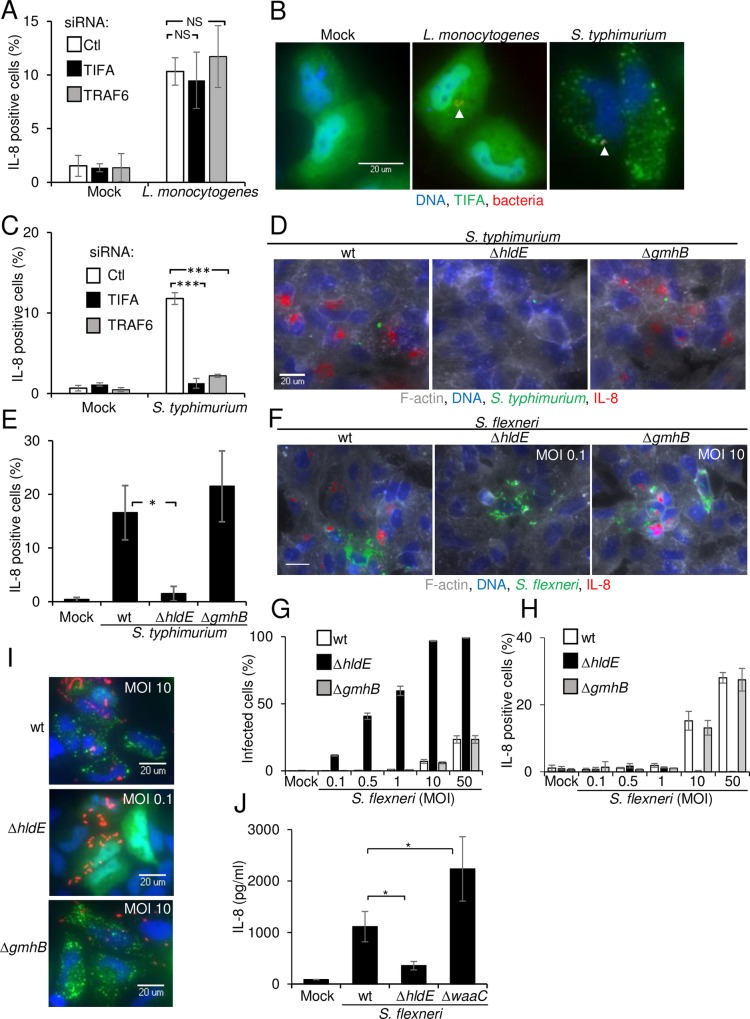
Sensing of HBP triggers TIFA/TRAF6-dependent innate immunity. **A**) TIFA and TRAF6 are not involved in *L*. *monocytogenes*-induced IL-8 production. Cells were transfected with control, TIFA- or TRAF6-targeting siRNAs, infected with *L*. *monocytogenes* for 3.5 hours and stained for IL-8. Data show the mean +/- SD of 3 independent experiments, ns: p>0.05. **B**) *S*. *typhimurium* infection induces TIFA oligomers. Hela cells were transfected with wild-type TIFA cDNA, infected with *L*. *monocytogenes* or *S*. *typhimurium* for 45 minutes and stained for TIFA (green) and DNA (blue). Arrows indicate bacteria (red). **C**) TIFA and TRAF6 are involved in IL-8 expression after *S*. *typhimurium* infection. Cells were transfected as in A, infected with *S*. *typhimurium* for 3.5 hours and stained for IL-8. Data show the mean +/- SD of 3 independent experiments, p***<0.0005. **D**) HBP is required for IL-8 induction after *S*. *typhimurium* infection. Cells were infected with wt, Δ*hldE* or Δ*gmhB S*. *typhimurium* (green) and stained for IL-8 (red), F-actin (grey) and DNA (blue). **E**) Quantification of IL-8 after infection with wt, Δ*hldE* or Δ*gmhB S*. *typhimurium*. Data show the mean +/- SD of 3 independent experiments, p*<0.05. **F**) HBP is required for IL-8 expression after *S*. *flexneri* infection. Cells were infected with wt, Δ*hldE* or Δ*gmhB S*. *flexneri* (green) and stained as in D. Scale bars, 20 μm. **G**) Comparison of the infection rates after infection with wt, Δ*hldE* or Δ*gmhB S*. *flexneri* at multiple MOIs. Data show the mean +/- SD of triplicate wells, graph representative of 3 independent experiments. **H**) Quantification of IL-8 after infection with wt, Δ*hldE* or Δ*gmhB S*. *flexneri*. Data show the mean +/- SD of triplicate wells, graph representative of 3 independent experiments. **I**) TIFA oligomerization is HBP-dependent. Cells were transfected with TIFA cDNA and infected with wt, Δ*hldE* or Δ*gmhB S*. *flexneri* (red). Cells were stained for TIFA (green) and DNA (blue). **J**) IL-8 secretion of *S*. *flexneri*-infected Caco-2 cells is largely HBP-dependent. ELISA assay measuring the secretion of IL-8 after infection of Caco-2 cells. Cells were infected for 6 hours with wt (MOI 400), Δ*hldE* (MOI 4) or Δ*waaC* (MOI 4) *S*. *flexneri*. Data correspond to the mean +/- SD of 3 independent experiments, p*<0.05.

To elucidate the mechanism triggering the activation of the TIFA/TRAF6 pathway, we tested whether TIFA was also involved in the induction of the IL-8 response observed after *Listeria monocytogenes* and *Salmonella typhimurium* infections. Like *S*. *flexneri*, these two enteroinvasive bacteria induce the secretion of the inflammatory cytokine IL-8. In both cases, IL-8 expression is potentiated via cell-cell communication between adjacent epithelial cells [13]. Depletion of neither TIFA nor TRAF6 had an impact on *L*. *monocytogenes*-induced IL-8 production (Fig 5A) and TIFA failed to form oligomers after infection (Fig 5B). In contrast, the depletion of either TIFA or TRAF6 abolished IL-8 expression after *S*. *typhimurium* infection (Fig 5C), while TIFA formed oligomers in both infected and bystander cells (Fig 5B). Since *S*. *flexneri* and *S*. *typhimurium* are both gram-negative, these results suggested that TIFA/TRAF6-dependent IL-8 expression was specifically triggered during gram-negative bacterial infections. We hypothesized that this innate immune response was induced by the recognition of HBP, a recently identified PAMP present in gram-negative bacteria [28]. HBP is a phosphorylated metabolic intermediate of lipopolysaccharide biosynthesis, produced from D-glycero-D-manno-heptose-7-phosphate by the HldE enzyme [28] (S4A Fig). The role of HBP in the induction of IL-8 expression was directly tested by measuring IL-8 production in response to infection with a *S*. *typhimurium* mutant deleted for the *hldE* gene (Δ*hldE*) and which expressed the dsRed protein under the *uhpT* promoter. Data showed that infection with the Δ*hldE* mutant, which is unable to synthesize HBP, failed to induce IL-8 production both in infected and bystander cells (Fig 5D and 5E). As expected, infection with bacteria deficient for the enzymes GmhB (Δ*gmhB*) or WaaC (Δ*waaC*), which act downstream of HldE in the ADP heptose biosynthetic pathway [30] (S4A Fig), induced strong IL-8 expression (Fig 5D and 5E, S4B Fig). The same experiment was repeated with *S*. *flexneri* mutants. Interestingly, the Δ*hldE* and Δ*waaC* mutants were dramatically more invasive than wild-type or Δ*gmhB* bacteria (Fig 5G and S4C Fig). However, at all multiplicities of infection tested, the absence of HBP led to a complete inhibition of IL-8 expression (Fig 5F and 5H). As with *S*. *typhimurium*, the Δ*gmhB* and Δ*waac* mutants induced massive IL-8 expression, indicating that the expression of IL-8 was dependent on bacterial synthesis of HBP (Fig 5F and 5H, S4D Fig). In addition, infection with the *S*. *flexneri* and *S*. *typhimurium* Δ*hldE* mutants failed to induce the oligomerization of TIFA (Fig 5I, S5A and S5B Fig). Finally, multiplex cytokine analysis showed that *S*. *flexneri* infection of HeLa cells induced the secretion of IL-6, IL-1β, IFNγ, IL-8 and TNFα in an HBP-dependent manner (S6A Fig). Furthermore, the induction of IL-8 and TNFα observed in Caco-2 cells after *S*. *flexneri* infection was also largely dependent on HBP (Fig 5J and S6B Fig). Altogether, these results show a causal link between HBP, the oligomerization of TIFA/TRAF6, the activation of NF-κB and inflammatory cytokine expression. They also show for the first time, that HBP is a critical PAMP that triggers inflammation in epithelial cells during infection by at least two invasive gram-negative pathogens, S. *typhimurium* and *S*. *flexneri*.

### ALPK1 controls *S*. *flexneri* infection-induced cytokine expression and TIFA oligomerization

**Fig 6:**
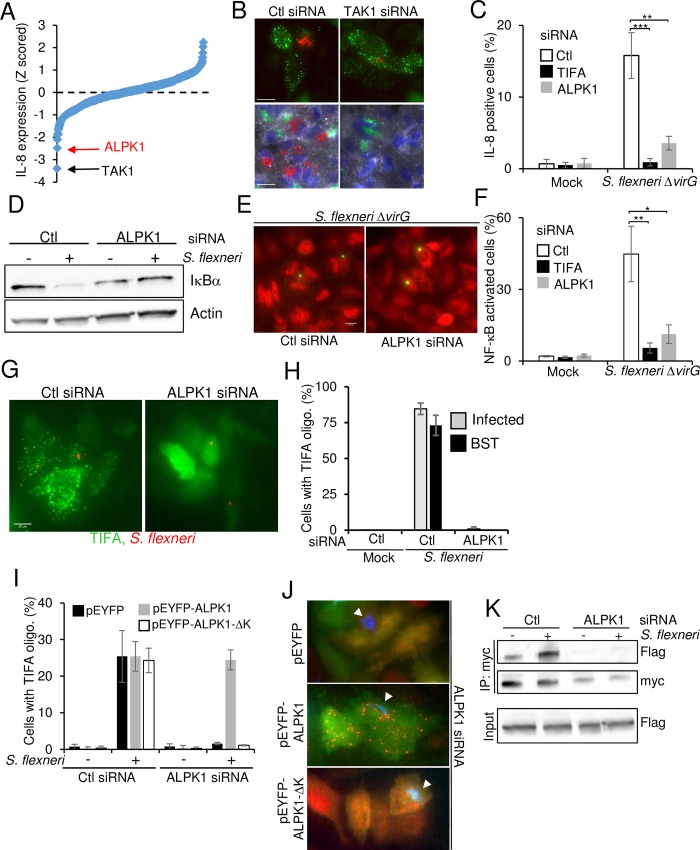
ALPK1 controls TIFA-mediated innate immunity during infection with invasive bacteria. **A**) Kinome RNAi screening data of IL-8 expression after *S*. *flexneri* infection in HeLa cells. IL-8 measurements were extracted with CellProfiler, Z-scored and ranked. **B**) Silencing TAK1 prevents *S*. *flexneri*-induced IL-8 expression but not TIFA oligomerization. Top panels show TIFA in green and *S*. *flexneri* in red. Bottom panels show F-actin in grey, DNA in blue, IL-8 in red and *S*. *flexneri* in green. Scale bars, 20 μm. **C**) Silencing ALPK1 inhibits IL-8 expression induced by *S*. *flexneri* infection. Cells were transfected with control, TIFA and ALPK1-targeting siRNAs, infected and stained for IL-8. Data correspond to the mean +/- SD of three independent experiments, p**<0.005, p***<0.0005. **D**) Silencing ALPK1 inhibits *S*. *flexneri*-induced IκBα degradation. Lysates of control or infected cells were blotted with IκBα or actin antibodies. **E**) ALPK1 depletion inhibits *S*. *flexneri-*induced NF-κB activation. Cells were transfected with control and ALPK1-targeting siRNAs, infected with *S*. *flexneri* Δ*virG* (green) and stained for NF-κB p65 (red). Scale bars, 20 μm. **F**) Silencing ALPK1 inhibits NF-κB activation induced by *S*. *flexneri* infection. Cells were transfected as in E, infected for 1h and stained for NF-κB p65. Cells showing NF-κB nuclear translocation were quantified. Data show the mean +/- SD of 3 independent experiments, p**<0.005, p*<0.05. **G**) Silencing ALPK1 prevents the formation of TIFA oligomers. Cells were transfected as in E and with wt TIFA cDNA. After infection (*S*. *flexneri* in red) for 45 minutes, cells were stained for TIFA (green). **H**) Impact of ALPK1 depletion on the formation of TIFA oligomers. Cells were treated as in G. Cells showing TIFA punctuates were manually quantified (n = 130 cells per condition), BST: bystander. Data show the mean +/- SD of triplicate wells, graph representative of 3 independent experiments. **I**) Only full length ALPK1 rescues TIFA oligomerization in ALPK1-depleted cells. Cells were transfected with control or ALPK1 siRNAs and then with pEYFP, pEYFP-ALPK1 or pEYFP-ALPK1-ΔK cDNA constructs. The fraction of cells showing TIFA oligomers was manually quantified (n>180 cells). Data show the mean +/- SD of three independent experiments. **J**) Images illustrating the oligomerization of TIFA in the rescue experiment as described in I. TIFA is show in red, YFP-ALPK1 in green and *S*. *flexneri* in blue. Arrows indicate bacteria. **K**) ALPK1 controls the TIFA-TRAF6 interaction. Co-immunoprecipitation of TIFA-myc after *S*. *flexneri* infection. HeLa cells were transfected with control or ALPK1 siRNAs and with myc-TIFA and Flag-TRAF6. They were then infected for 1 hour at MOI 10. Myc IPs were blotted with anti-Flag and anti-myc antibodies and input lysates with an anti-Flag antibody.

The observation that TIFA oligomerization was dependent on T9 and the FHA domain of TIFA suggested that at least one kinase was involved upstream of TIFA to control IL-8 expression. In order to identify kinase candidates, an RNAi screen targeting each gene of the human kinome with three individual siRNAs, was performed. TAK1, known to be involved in *S*. *flexneri*-induced NF-κB activation downstream of TRAF6 and RIPK2 [8], was the strongest negative hit (S2 Table, Fig 6A). We tested whether this kinase could also control TIFA oligomerization during infection. Although depleting TAK1 completely abrogated IL-8 production (Fig 6A and 6B, S2 Table), TIFA oligomers were still visible in infected and bystander cells (Fig 6B), confirming that TAK1 was implicated downstream of TIFA. The second strongest hit was ALPK1. ALPK1 belongs to the atypical kinase group [31] and is poorly characterized. It is a component in apical transport of epithelial cells [32]. Furthermore, polymorphism in the *alpk1* gene is associated with type 2 diabetes, dyslipidemia, gout and chronic kidney disease [33–36]. Strikingly, the *alpk1* and *tifa* genes are direct neighbors on human chromosome 4 [37], suggesting that they may be co-regulated and part of a common cellular pathway. ALPK1 was thus further investigated for its implication in *S*. *flexneri* infection and TIFA-dependent innate immunity. First, the role of ALPK1 in IL-8 production after *S*. *flexneri* infection was confirmed by intracellular IL-8 staining (Fig 6C) and ELISA (S7A Fig). The secretion of IL-6, IL-1β, IFNγ and TNFα was reduced in ALPK1-depleted cells (S7B Fig), showing that ALPK1 is a master regulator of *S*. *flexneri*-induced inflammatory cytokine expression, a process largely triggered in response to HBP (S6A Fig). Since TIFA and TRAF6 regulated *S*. *flexneri*-induced NF-κB activation, we investigated the role of ALPK1 in this process. Western blot experiments performed on uninfected and infected cells revealed that depleting ALPK1 reduced the degradation of the inhibitor of NF-κB, IκBα, in infected cells (Fig 6D). In agreement, ALPK1 depletion also impaired the nuclear translocation of NF-κB after *S*. *flexneri* infection without significantly affecting bacterial entry (Fig 6E and 6F and S2 Fig). Altogether, these results suggested that ALPK1 was a promising candidate for the control of TIFA-dependent innate immunity. The role of ALPK1 in this process was directly addressed by several means. First, depletion of ALPK1 prevented the formation of TIFA oligomers both in infected and bystander cells during *S*. *flexneri* infection (Fig 6G and 6H). Second, in rescue experiments, whereby cells were transfected with control or ALPK1 siRNA and then transfected with the empty vector pEYFP or a siRNA-resistant full-length ALPK1-YFP cDNA construct (Fig 6I and 6J), overexpression of YFP-ALPK1 did not induce the formation of TIFA oligomers in the absence of infection, indicating that this process was tightly regulated. Notably, when ALPK1-depleted cells were transfected with full-length YFP-ALPK1, TIFA oligomerization was restored in a large fraction of infected and bystander cells. This result excluded the possible contribution of RNAi off target effects and unambiguously established the role of ALPK1 in *S*. *flexneri*-induced TIFA oligomerization. Interestingly, transfection of a siRNA-resistant cDNA construct deleted for the kinase domain of ALPK1 (YFP-ALPK1-ΔK) failed to rescue TIFA oligomerization (Fig 6I and 6J), showing that the kinase domain of ALPK1 was necessary for the induction of TIFA oligomerization after *S*. *flexneri* infection. Finally, the role of ALPK1 on the TIFA-TRAF6 interaction was investigated by co-immunoprecipitation experiments. Data showed that the TIFA-TRAF6 interaction induced upon *S*. *flexneri* infection was strongly reduced in ALPK1-depleted cells, demonstrating that this interaction was ALPK1-dependant (Fig 6K). Altogether, these results showed that ALPK1 is a master regulator of cytokine expression during *S*. *flexneri* infection and that TIFA oligomerization depends on the kinase domain of ALPK1.

### ALPK1 is a master regulator of HBP-induced innate immunity

**Fig 7:**
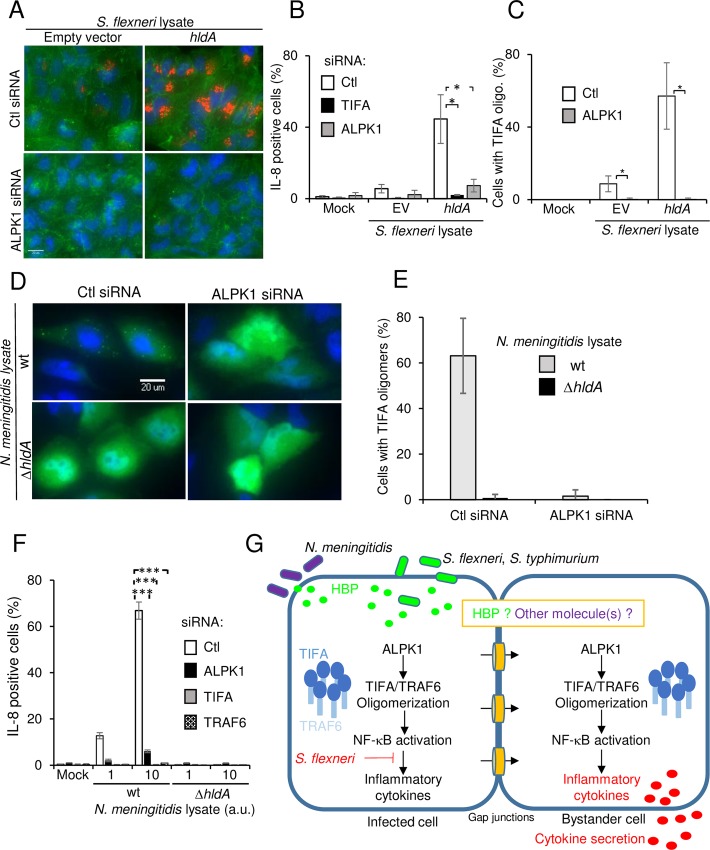
ALPK1 is a master regulator of HBP-induced innate immunity. **A**) ALPK1 controls IL-8 expression and the formation of TIFA oligomers induced by HBP. Cells were transfected with control or ALPK1 siRNAs and incubated with lysates from *S*. *flexneri* containing pUC19 empty vector (EV) or expressing *hldA* from pUC19. They were stained for IL-8 (red), F-actin (green) and DNA (blue). Scale bars, 20 μm. **B**) Quantification of data shown in F. Data show the mean +/- SD of 3 independent experiments, p*<0.05. **C**) Silencing ALPK1 prevents HBP-induced TIFA oligomerization. Cells were transfected as in A and with wt TIFA. After incubation with lysates from *S*. *flexneri* containing pUC19 or pUC19-*hldA*, cells were stained for TIFA. Cells showing TIFA punctuates were manually quantitated (n = 105 cells per condition). Data show the mean +/- SD of 3 independent experiments, p*<0.05. **D**) Silencing ALPK1 abrogates the formation of TIFA oligomers induced by *N*. *meningitidis* HBP. HeLa cells were transfected with control or ALPK1-targeting siRNA and then with wt TIFA cDNA. After incubation with lysates from wt or Δ*hldA N*. *meningitidis*, cells were stained for TIFA (green) and DNA (blue). **E**) Impact of ALPK1 depletion on TIFA oligomerization. Cells were treated as in D. The fraction of cells showing TIFA punctuates was manually quantitated with n = 130 cells per condition. Data show the mean +/- SD of triplicate wells and the graph is a representative of 3 independent experiments. **F**) Silencing ALPK1 abrogates the production of IL-8 induced by *N*. *meningitidis* lysates. Cells were transfected with control, TIFA, TRAF6 or ALPK1-targeting siRNA. Cells were then incubated with lysates from wt or Δ*hldA N*. *meningitidis* and stained for IL-8. Data show the mean +/- SD of triplicate well and the graph is a representative of 3 independent experiments, p***<0.0005. **G**) Schematic illustration of the ALPK1/TIFA/TRAF6 pathway controlling IL-8 expression after infection by gram-negative bacteria.

As *S*. *flexneri*-induced TIFA oligomerization occurred in response to HBP (Fig 5I), we tested whether ALPK1 was involved in this process. Cells were stimulated with lysates from *S*. *flexneri* containing an empty pUC19 vector or expressing the HBP-synthesizing enzyme HldA from *N*. *meningitidis* [28]. As expected, the lysate from HldA-overexpressing bacteria was more potent at inducing IL-8 expression than those of wild-type bacteria (Fig 7A and 7B). Interestingly, depletion of ALPK1 prevented the oligomerization of TIFA (Fig 7C) as well as IL-8 production (Fig 7A and 7B) in response to both lysates, showing that ALPK1 controlled the oligomerization of TIFA following HBP recognition. As with TIFA, depletion of ALPK1 failed to inhibit IL-8 expression and NF-κB activation observed after *L*. *monocytogenes* infection (S8A and S8B Fig), suggesting a specific implication in infection by invasive gram-negative bacteria. Furthermore, ALPK1 was not required to activate NF-κB in response to PMA (S9A Fig) or TNFα (S9B Fig). As with TIFA and TRAF6, depleting ALPK1 had a moderate but significant effect on C12-iE-DAP-induced NF-κB activation (S9C Fig). The role of ALPK1 was further characterized in the inflammatory response triggered by *Neisseria meningitidis*, an important gram-negative extracellular human pathogen. This bacterium is responsible for meningitis and other forms of meningococcal diseases including meningococcemia, a case of life-threatening sepsis [38]. Upon infection with this pathogen, HBP can be secreted or released by lysing bacteria [28]. We confirmed that treating HeLa cells with *N*. *meningitidis* lysate induced TIFA oligomerization (Fig 7D and 7E) and IL-8 expression (Fig 7F). Furthermore, depleting either TIFA or TRAF6 prevented IL-8 expression (Fig F). Interestingly, we found that TIFA oligomerization and IL-8 expression were both completely abrogated in ALPK1-depleted cells (Fig 7D, 7E and 7F), showing that ALPK1 also controls the innate immune response to *N*. *meningitidis* infection (Fig 7G). Altogether, these results show that HBP is a key bacterial PAMP sensed by epithelial cells during infection by both invasive and extracellular gram-negative bacteria and that TIFA/TRAF6-dependent innate immunity against HBP is controlled by ALPK1.

## Discussion

An RNAi screen implicated TIFA and TRAF6 in the control of IL-8 expression after *S*. *flexneri* infection. We show that these two proteins act upstream of NF-κB p65 activation in infected and bystander cells. In particular, we provide evidence demonstrating that *S*. *flexneri* induces the oligomerization of TIFA and TRAF6 in infected and bystander cells in a FHA/T9-dependent manner. In cells expressing a TIFA mutant unable to bind TRAF6, the formation of TRAF6 oligomers was not observed, showing that the TIFA-TRAF6 interaction is necessary to trigger TRAF6 oligomerization. Given that TRAF6 oligomerization has been shown to increase its E3 ubiquitin ligase activity [39], our data suggest that TIFA works as an adaptor protein promoting TRAF6 oligomerization and thereby NF-κB activation and inflammatory gene expression (Fig 7G). In infected and bystander cells, TIFA oligomers are distributed evenly throughout the cytoplasm. They appear within minutes of infection and are still visible four hours post infection. Co-staining of TIFA and lysosomal-associated membrane protein 1 (LAMP1) in *S*. *flexneri*-infected cells revealed that TIFA/TRAF6 oligomers are not localized to lysosomes (S10 Fig). More work is needed to determine whether these aggregation platforms are associated with other subcellular structures or whether they freely diffuse in cells.

We show that during *S*. *flexneri* and *S*. *typhimurium* infection, the TIFA/TRAF6 pathway is activated in response to the bacterial monosaccharide HBP, present in gram-negative bacteria. Indeed, we found that the Δ*hldE* mutants of *S*. *flexneri* and *S*. *typhimurium*, which are unable to synthesize HBP, fail to induce the oligomerization of TIFA and the production of IL-8. These results open up a new avenue to understand the molecular processes controlling inflammation in bacterial infection and highlight the central role of HBP during infection by invasive bacteria. In contrast to the study by Gaudet *et al*. [28], the production of IL-8 in response to *S*. *flexneri* and *S*. *typhimurium* infection is unlikely due to the simple mechanism of HBP endocytosis. Indeed, we previously demonstrated that noninvasive *S*. *flexneri* bacteria failed to induce IL-8 expression [13]. This point was further confirmed by Lippmann *et al*. who showed that the expression of IL-8 in bystander cells requires bacterial internalization [21]. Mechanisms explaining how HBP could therefore be detected within minutes of bacterial invasion have to be envisioned. Although there is, to our knowledge, no evidence in the literature for the release of metabolites via type III secretion, we cannot exclude the possibility that HBP may be directly secreted into the host cytoplasm via the injectisome. An alternative mechanism would consist in the cellular uptake of HBP during the process of bacterial internalization. A study using dynamic imaging and advanced large volume correlative light electron microscopy recently reported that two distinct compartments are formed during the first step of bacterial invasion: the bacterial containing vacuole (BCV) and surrounding macropinosomes [40]. Whereas the membrane of the BCV tightly surrounds the bacterium, macropinosomes are heterogeneous in size and contain significant volumes of extracellular fluid [40]. HBP, released from residual secretion or bacterial lysis, may be engulfed by infected cells via the BCV or macropinosomes and released into the cytoplasm shortly after membrane rupture. The small molecular size of HBP (370 Da) should allow its diffusion to adjacent cells via gap junctions leading to TIFA oligomerization and IL-8 expression in bystander cells (Fig 7G). Alternatively, HBP sensing in infected cells may lead to the production of a second messenger that could diffuse to bystander cells and activate the ALPK1/TIFA/TRAF6 pathway. In the case of *S*. *typhimurium*, the complete rupture of the internalization vacuole is a rare event. In most cases, bacteria remain inside *Salmonella*-containing compartments. Interestingly, a recent study shows that early *Salmonella*-containing compartments are leaky and that autophagy proteins promote the repair of endosomal membranes damaged by the type III secretion system 1 [41]. In this context, HBP may leak out of these early compartments, be released into the cytoplasm of infected cells and induce IL-8 expression both in infected and bystander cells, as observed previously [13]. We showed that secretion of inflammatory cytokines after *S*. *flexneri* infection of epithelial cells *in vitro* is largely HBP-dependent, which supports a central role of HBP in the control of innate immunity in *S*. *flexneri* infection. More work is needed to determine the exact contribution of HBP in *in vivo* infection where other PAMPs, including peptidoglycan-derived peptides and LPS, have previously been shown to play a role [4, 42].

Our results show that TIFA’s activity in *S*. *flexneri*-induced IL-8 expression is dependent on residue T9 and the FHA domain of TIFA. As the interaction between these two features occurs once T9 is phosphorylated and is required to trigger TIFA oligomerization, we searched for a kinase acting upstream of TIFA oligomerization in bacterial infection. We identified the kinase ALPK1 in a human kinome RNAi screen. Strikingly, the genes coding for ALPK1 and TIFA are immediate neighbors on human chromosome 4 [37]. Gene neighborhood is conserved across several species including coelacanth, xenopus, chicken and mouse, suggesting that both genes may be co-regulated and the encoded proteins part of a same cellular pathway. We show that depleting ALPK1 strongly reduced NF-κB activation and the production of several cytokines including IL-8, TNFα, IL-1β, IFNγ and IL-6 after *S*. *flexneri* infection. IL-8 production was also reduced after *S*. *typhimurium* infection. ALPK1 depletion completely prevented the formation of TIFA oligomers after *S*. *flexneri* infection, a process triggered in response to HBP sensing. TIFA oligomerization was restored by overexpressing a siRNA-resistant full length ALPK1 construct. In contrast, overexpressing a construct deleted of the kinase domain of ALPK1 failed to do so, showing that the kinase domain of ALPK1 is essential for the regulation of TIFA oligomerization. In addition, co-immunoprecipitation experiments revealed that the TIFA-TRAF6 interaction is dependent on ALPK1. All these results demonstrate that ALPK1 is involved in the early signaling cascade controlling inflammation following cellular invasion by gram-negative bacterial pathogens. Furthermore, we show that ALPK1 is also implicated in the control of inflammation after stimulation with *N*. *meningitidis* lysates, indicating that this kinase acts as a master regulator of innate immunity to both invasive and extracellular gram-negative bacteria. ALPK1 is an atypical kinase belonging to the α-kinase family that recognizes phosphorylation sites in the context of an alpha-helical conformation [31]. The fact that T9 is not in this environment is not sufficient to exclude that ALPK1 can directly phosphorylate TIFA. Indeed, it has been shown that members of this protein family can also phosphorylate substrates independently of a helical conformation [31]. More experiments are required to elucidate the mode of action of ALPK1 in the activation of the TIFA/TRAF6 pathway. In addition, it will be informative to determine whether HBP can directly bind to ALPK1 or whether this new bacterial PAMP binds to a yet unknown pathogen recognition receptor able to activate ALPK1 and trigger TIFA oligomerization. Interestingly, by sensing the presence of HBP, a metabolite of the LPS biosynthetic pathway, such a receptor would constitute a new specific sensor for the presence of gram-negative bacteria.

In conclusion, we show that ALPK1 is a master regulator of innate immunity against both invasive and extracellular gram-negative bacteria. This kinase acts in response to the detection of HBP to activate the TIFA/TRAF6 pathway. By regulating the expression of inflammatory cytokines, this new signaling pathway is critical to orchestrate the initial host immune response and limit bacterial dissemination within infected tissues. It may also contribute to the control of intestinal homeostasis by regulating the molecular cross-talk taking place between gram-negative bacteria of the microbiota, the intestinal epithelium and the immune system.

## Materials and methods

### Cell culture, transfections and cDNA constructs

HeLa (American Type Culture Collection) and HEK293 (American Type Culture Collection) cells were cultured in Dulbecco’s modified Eagle’s (DMEM) medium supplemented with 10% FCS and 2 mM Glutamax-1. Caco-2 cells (American Type Culture Collection) were cultured in MEM, 20% FCS and 1% non-essential amino acids. Transfection of siRNAs was carried out using RNAiMAX (Invitrogen). HeLa cells, seeded in 96-well plates (6,000 cells/well), were reverse transfected with 20 nM siRNA according to the manufacturer’s instruction. Cells were used 72 hours after transfection. siRNAs against TIFA (s40984), TRAF6 (s14389) and ALPK1 (s37074) were from Ambion and TAK1 from Dharmacon. For cDNA transfection, HeLa cells were seeded in a 96-well plate at a density of 12,500 cells/well. The next day, cells were transfected with 80 ng of plasmid using Fugene 6 (Roche) according to the manufacturer’s instruction. Wild-type, T9A, E178A and the RKN TIFA cDNA constructs [27] were kindly provided by Prof. M.D. Tsai (Institute of Biological Chemistry, Academia Sinica, Taiwan). They were made TIFA siRNA (s40984) resistant by the introduction of 3 silent point mutations within the recognition site of the siRNA. Point mutations were introduced by overlapping PCR using primers TIFA_BamHI_F, TIFA_R2, TIFA_F2, TIFA_XbaI_R and TIFA_EA_XbaI_R (listed in S3 Table). The resulting PCR products were digested with BamHI and XbaI and ligated into pcDNA3. A YFP-ALPK1 construct was kindly provided by Pr R. Jacob (Marburg University, Germany). It was made siRNA (s37074)-resistant by the introduction of 5 silent point mutations at positions 761-762-763-767-768 by directed mutagenesis (Agilent Technology). A mutant deleted for the kinase domain of ALPK1 was generated by introducing a stop codon at position 3059 before the kinase domain by directed mutagenesis. All primers used in directed mutagenesis are listed in S3 Table.

For TIFA and ALPK1 rescue experiments, Hela cells were first reverse transfected with TIFA or ALPK1 siRNAs (s40984, s37074 respectively). After 48 hours, they were transfected with the different siRNA-resistant TIFA cDNA constructs or siRNA-resistant full length or kinase domain-deleted YFP-ALPK1. As a negative control, cells were transfected with the empty vectors pcDNA or pEYFP. Wild-type Flag-TRAF6 cDNA was a gift from John Kyriakis (Addgene plasmid # 21624) [43].

### Bacterial strains

The M90T wild-type *Shigella flexneri* strain and the *icsA* (*virG*) deletion mutant have been previously described [44]. The *Salmonella typhimurium* 12023 strain expressing pKD46 was provided by J. Guignot (Institut Cochin, Paris, France) and the EGDe.PrfA *Listeria monocytogenes* strain stably expressing GFP [45] was provided by Prof. P. Cossart (Institut Pasteur, Paris, France). All *Shigella* and *Salmonella* strains were transformed with the pMW211 plasmid and constitutively express the dsRed protein [13]. When specifically mentioned, bacteria were alternatively transformed with a variant of pMW211 expressing dsRed under the control of the *uhpT* promoter (P*uhpT*::dsRed) [17]. For *Neisseria meningitidis*, a piliated capsulated Opc^-^ Opa^-^ variant of serogroup C strain 8013 named 2C43 was used. The *hldA* deficient mutant was obtained as previously described in [46].

### Deletion mutants and overexpressing strains

*S*. *flexneri* M90T and *S*. *typhimurium* 12023 deletion mutants were generated by allelic exchange using a modified protocol of lambda red-mediated gene deletion [47]. Briefly, to obtain the *S*. *flexneri* M90T and *S*. *typhimurium hldE* (Δ*hldE*), *gmhB (*Δ*gmhB)* and *waaC* (Δ*waaC)* deletion mutants, the kanamycin cassette of the pkD4 plasmid was amplified by PCR with the primers listed in S3 Table. The purified PCR product was electroporated into the wild-type strains expressing the genes for lambda red recombination from the pKM208 (for *S*. *flexneri* mutants) or pKD46 (for *S*. *typhimurium* mutants) plasmids [48]. Recombinants were selected on TSB or LB plates containing 50 μg ml-1 of kanamycin. Single colonies were screened by PCR.

*S*. *flexneri* M90T overexpressing the *hldA* gene from *Neisseria meningitidis* was generated as follows. The *hldA* gene was amplified by PCR from a bacterial lysate with the primers listed in S3 Table. After gel purification (Macherey-Nagel), the PCR product was digested with EcoRI and HindIII, and ligated into EcoRI/HindIII-digested pUC19 (Life Technology). The ligation product was used to transform Top10 *E*. *Coli*. pUC19-HldA was purified and used to electroporate *S*. *flexneri* M90T. As a control, *S*. *flexneri* M90T was also electroporated with the pUC19 empty vector.

### Preparation of bacterial lysates

Bacterial lysates were prepared as described in Gaudet *et al*. [28]. Briefly, 1 OD_600_ unit of bacteria from an overnight culture was centrifuged, resuspended in 100 μl PBS and boiled for 15 mins. Bacterial debris were removed by centrifugation at 13 000 rpm for 10 mins. Supernatants were collected and protein concentration was measured by BCA assay (Interchim) for normalization. Lysates were then treated with RNAse A (10 μg/ml), DNAse I (20U) (both Roche) and proteinase K (100 μg/ml) (Sigma-Aldrich). Samples were boiled for a further 5 minutes, centrifuged and the supernatant passed through a 0.22 μm filter. Lysates were stored at -20°C.

### Infection assays and stimulations

*S*. *flexneri*, *S*. *typhimurium* and *L*. *monocytogenes* were used in exponential growth phase. *Shigella* and *Salmonella* were coated, or not, with poly-L-lysine prior to infection. Cells seeded in 96-well plates, were infected at indicated MOIs in DMEM supplemented with 10 mM Hepes and 2 mM glutamax-1. After adding bacteria, plates were centrifuged for 5 minutes and placed at 37°C for indicated time periods. Extracellular bacteria were killed by gentamicin (100 μg/ml). For stimulation experiments, cells were incubated with PMA (Sigma), C12-iEDAP (Invivogen) and TNFα (R&D Systems) at indicated concentrations. For intracellular IL-8 measurements, monensin (50 μM) was added together with gentamycin to block IL-8 secretion. Infection and stimulation assays were stopped by 4% PFA fixation.

### RNAi screens

The screening methodology has already been described [17]. Briefly, RNA interference (RNAi) directed against the human genome was achieved using the commercially available genome-wide siRNA library from Dharmacon (pools of 4 siRNAs/gene). The human kinome RNAi screen was performed with the Ambion library made of three individual siRNAs per gene. All experiments were conducted in a 384-well plate format. In addition to gene-specific siRNAs, all plates contained general siRNA controls for transfection efficiency (e.g. Kif11), positive control siRNAs known to affect inflammation after *S*. *flexneri* infection (TAK1, p65 NF-κB) and non-targeting siRNAs. In each experiment, 25 μl of RNAiMAX/DMEM (0.1 μl/24.9 μl) mixture was added to each well of the screening plates containing 1.6 pmol siRNA diluted in 5 μl RNase-free ddH2O. Screening plates were incubated at room temperature (RT) for 1 hour. Following incubation, 600 HeLa CCL-2 cells were added per well in a volume of 50 μl DMEM/16% FCS, resulting in a final FCS concentration of 10%. Plates were incubated at 37°C and 5% CO2 for 72 h prior to infection. For infection, *S*. *flexneri* M90T Δ*virG* pCK100 (PuhpT::dsRed) were harvested in exponential growth phase and coated with 0.005% poly-L-lysine (Sigma-Aldrich). Afterwards, bacteria were washed with PBS and resuspended in assay medium (DMEM, 2 mM L-Glutamine, 10 mM HEPES). 20 μl of bacterial suspension was added to each well with a final MOI of 15. Plates were then centrifuged for 1 min at 37°C and incubated at 37°C and 5% CO_2_. After 30 min of infection, 75 μl were aspirated from each well and monensin (Sigma) and gentamicin (Gibco) were added to a final concentration of 66.7 μM and 66.7 μg/ml, respectively. After a total infection time of 3.5 hours, cells were fixed with 4% PFA for 10 minutes. Liquid handling was performed using the Multidrop 384 (Thermo Scientific) for dispension steps and a plate washer (ELx50-16, BioTek) for aspiration steps. For immunofluorescent staining, cells were washed with PBS using the Power Washer 384 (Tecan). Subsequently, cells were incubated with a mouse anti-human IL-8 antibody (1:300, BD Biosciences) in staining solution (0.2% saponin in PBS) for 2 hours at RT. After washing the cells with PBS, Hoechst (5 μg/ml, Invitrogen), DY-495-phalloidin (1.2 U/ml, Dyomics) and Alexa Fluor 647-coupled goat anti-mouse IgG (1:400, Invitrogen) were added and incubated for 1 hour at RT. The staining procedure was performed using the Biomek NXP Laboratory Automation Workstation (Beckman Coulter).

Microscopy was performed with Molecular Devices ImageXpress microscopes. MetaXpress plate acquisition wizard with no gain, 12 bit dynamic range, 9 sites per well in a 3×3 grid with no spacing and no overlap and laser-based focusing was used. Robotic plate handling was used to load and unload plates (Thermo Scientific). A 10X S Fluor objective with 0.45NA was used.

Data analysis was performed using the computational infrastructure described in [17]. Cell counts, rates of infection and IL-8 positive cells were quantified as described in [17]. In brief, intensity and texture features were extracted from bacterial and IL-8 images. Based on these features, cells were scored for infection and IL-8 expression using CellClassifier and supervised machine learning using a Support Vector Machine based binary classifier [49]. Measurements were normalized for plate-to-plate variations and population context dependency as described in [17].

### Immunofluorescence

After fixation, cells were permeabilized in 0.1% Triton X-100 for 10 minutes, incubated in PBS supplemented with 0.5% BSA for 2 hours and then overnight at 4°C with different combinations of primary antibodies. NF-κB p65 localization was visualized by using a mouse monoclonal anti-p65 antibody (Santa Cruz Biotechnology, USA), TIFA was visualized with a polyclonal rabbit anti-TIFA primary antibody (Sigma-Aldrich), and LAMP1 was visualized with an anti-mouse anti-LAMP1 (Abcam). Cells were then stained with Alexa 647- or Alexa 488-conjugated secondary antibodies (Invitrogen, Carlsbad, USA). DNA and F-actin were stained with Hoechst and FITC-phalloidin, respectively. The production of IL-8 was measured by immunofluorescence using an anti-human IL-8 antibody in 0.2% saponin in PBS (BD Pharmingen, San Jose, USA) 4 hours post infection.

### Automated microscopy and image analysis

Images were automatically acquired with an ImageXpress Micro (Molecular devices, Sunnyvale, USA). Image analysis was performed using the custom module editor (CME) of MetaXpress. Briefly, cell nuclei were identified by the "autofind blobs" function of the CME. Nuclei were then extended by 6 pixels to define the cellular mask of each cell that was used to measure bacteria and IL-8 signals. Bacteria and IL-8 signals were both detected with the "keep marked object" function of the CME based on minimal/maximal size requirements and intensity threshold. Cells showing IL-8 signals above threshold were defined as IL-8 positive. Quantification of NF-κB activation was performed with the "translocation enhanced" module of MetaXpress (Molecular Devices, USA) that automatically identifies the nuclei and cytoplasmic compartments from a Hoechst image. Quantification was done by measuring the intensity ratio of p65 in the nucleus and the cytoplasm in several thousand cells per well and in three wells per condition. Cells showing nuclear/cytoplasmic p65 intensity ratio above a threshold ratio were defined as NF-κB positive cells.

### Cell lysis, immunoprecipitation and immunoblotting

Cells were plated in 6-well plates (180 000 cells/well), transfected or not with 20 nM siRNA and/or 2.4 μg cDNA and infected according to the experiment. After infection, cells were washed twice in ice cold PBS with gentamicin (100 μg/ml), lysed in RIPA buffer supplemented with inhibitors of proteases (Promega) and phosphatases (Thermofisher Scientific), incubated on ice for 30 minutes and subsequently centrifuged at 4°C for 30 minutes at 16,000g. The BCA Protein Assay kit (Interchim) was used to determine protein concentration. 15–20 ug of protein was subjected to SDS-polyacrylamide gels and electroblotted onto nitrocellulose membranes. For immunoprecipitation (IP), cell lysates were incubated with an anti-myc antibody (9E10, Santa Cruz) overnight. Protein A/G-coated beads (ThermoFisher) were then added for 2 hours and washed six times in Mac Dougall buffer. Cell lysates and IPs were diluted in Laemmli buffer containing SDS and β-mercaptoethanol, boiled for 6 minutes and subjected to SDS-PAGE. Immunoblotting was performed using primary antibodies diluted in phosphate buffered saline containing 0.1% Tween and 5% nonfat dry milk. HRP-conjugated secondary antibodies were purchased from GE Healthcare or Cell signaling technology or ThermoFisher Scientific. The blots were developed with an enhanced chemiluminescence method (SuperSignal West Pico Chemiluminescent substrate, Thermofisher Scientific).

### ELISA and cytokine multiplex assays

IL-8 secretion was measured by ELISA in the supernatant of HeLa and Caco-2 cells infected with *S*. *flexneri* for 6 hours. The cell-free supernatants from triplicate wells were analyzed for their IL-8 content using the commercial ELISA kit (eBioscience). The secretion of additional cytokines including TNFα, IL-1β, IL-6 and IFNγ was measured using the Cytokine Human Magnetic 10-plex Panel for Luminex Platform (Life Technologies).

### Statistical analysis

Data are expressed as mean ± standard deviation of triplicates samples as indicated. p values were calculated with a two-tailed two-sample equal variance t-test.

## Supporting information

S1 Fig Images illustrating the RNAi screen assay. HeLa cells were infected for 3.5 hours with *S*. *flexneri* Δ*virG* expressing dsRed under the control of the *uhpT* promoter (in green). After fixation, cells were stained for F-actin (in grey), DNA (in blue) and IL-8 (in red). Scale bars, 20 μm.
(PDF)

S2 Fig Silencing TIFA or ALPK1 has no significant effect on *S. flexneri* entry. HeLa cells were transfected for 72 hours with control, TIFA- or ALPK1-targeting siRNAs. HeLa cells were infected for 3.5 hours with Δ*virG S*. *flexneri* expressing dsRed. After fixation, cells were stained for F-actin and DNA. Infection rate was evaluated by automated image analysis. Data correspond to the mean +/- SD of 3 independent experiments, NS = non-significant p>0.05.
(PDF)

S3 Fig TIFA oligomerization after *S*. *flexneri* infection of Caco-2 cells. Caco-2 cells were transfected with a wild-type TIFA cDNA construct and infected for 3 hours with Δ*virG S*. *flexneri* expressing dsRed (in blue). After fixation, cells were co-stained for TIFA (in green) and NF-κB p65 (in red). Scale bars, 20 μm.
(PDF)

S4 Fig *S*. *typhimurium* and *S*. *flexneri*-induced IL-8 production is largely HBP-dependent. **A**) ADP-Heptose biosynthetic pathway. **B**) *S*. *typhimurium*-induced IL-8 production is largely HBP-dependent. HeLa cells were infected with *S*. *typhimurium* for 4 hours, fixed and stained for intracellular IL-8. Data correspond to the mean +/- SD of three independent experiments, p**<0.005. **C**) Infectivity of wt, Δ*hldE* and Δ*waaC S*. *flexneri*. HeLa cells were infected with wt (MOI 100), Δ*hldE* (MOI 1) and Δ*waaC* (MOI 1) *S*. *flexneri* expressing dsRed. The rate of infection was quantified automatically. Data correspond to the mean +/- SD of three independent experiments, non-significant p>0.05. **D**) *S*. *flexneri*-induced IL-8 production is largely HBP-dependent. HeLa cells were infected with *S*. *flexneri* for 4 hours, fixed and stained for intracellular IL-8. Data correspond to the mean +/- SD of three independent experiments, p**<0.005.
(PDF)

S5 Fig TIFA oligomerization is HBP-dependent. **A**) HeLa cells were transfected with a wild-type TIFA cDNA construct. After 24 hours, they were infected with wild-type, Δ*hldE* or Δ*gmhB S*. *typhimurium* expressing dsRed under the control of the *uhpT* promoter. The fraction of infected cells showing TIFA punctuates was manually evaluated. Data correspond to the mean +/- SD of 3 independent experiments. **B**) HeLa cells were transfected with a wild-type TIFA cDNA construct. After 24 hours, they were infected with wild-type, Δ*hldE* or Δ*gmhB S*. *flexneri* expressing dsRed. The fraction of infected cells showing TIFA punctuates was manually evaluated. Data correspond to the mean +/- SD 3 independent experiments.
(PDF)

S6 Fig The production of cytokines induced by *S. flexneri* infection is largely HBP-dependent. **A**) HeLa cells were infected or not with *S*. *flexneri* for 6 hours with wt (MOI 10), Δ*hldE* (MOI 0.1) and Δ*waaC* (MOI 0.1) *S*. *flexneri*. Cytokine secretion was measured in the supernatant of infected cells by a multiplex cytokine assay. Data correspond to the mean +/- SD of triplicates, p**<0.005, p***<0.0005. # indicates not detected. **B**) Caco-2 cells were infected or not with *S*. *flexneri* for 6 hours with wt (MOI 10), Δ*hldE* (MOI 0.1) and Δ*waaC* (MOI 0.1) *S*. *flexneri*. Cytokine secretion was measured in the supernatant of infected cells by a multiplex cytokine assay. Data correspond to the mean +/- SD of triplicates, p**<0.005. IL-1β, IFNγ and IL-6 were not detected in Caco-2 cells.
(PDF)

S7 Fig The production of cytokines induced by *S. flexneri* infection depends on ALPK1. **A**) ELISA assay showing that *S*. *flexneri*-induced IL-8 expression is ALPK1-dependent. HeLa cells were transfected with control or ALPK1 siRNA, and infected, or not, with *S*. *flexneri* for 6 hours. IL-8 secretion was measured in the supernatant of infected cells by ELISA. Data correspond to the mean +/- SD of three independent experiments, p*<0.05. **B**) HeLa cells were transfected with control or ALPK1 siRNA, and infected or not with *S*. *flexneri* for 6 hours. Cytokine secretion was measured in the supernatant of infected cells by a multiplex cytokine assay. Data correspond to the mean +/- SD of triplicates, p**<0.005, p***<0.0005. # indicates not detected.
(PDF)

S8 Fig ALPK1 is not involved in *L. monocytogenes*-induced inflammation. **A**) ALPK1 is not involved in *L*. *monocytogenes*-induced IL-8 expression. HeLa cells were transfected for 72 hours with control or ALPK1-targeting siRNAs and infected for 3.5 hours with *L*. *monocytogenes* expressing GFP. After fixation, cells were stained for F-actin, DNA, and IL-8. IL-8 was quantified by automated image analysis. Data correspond to the mean +/- SD of triplicate wells and the graph is representative of 3 independent experiments, ns: non-significant p>0.05. **B**) ALPK1 is not involved in *L*. *monocytogenes*-induced NF-κB activation. Cells were treated as in A but stained for NF-κB p65 after one hour of infection. Quantification of the NF-κΒ p65 nuclear/cytoplasmic fluorescence intensity ratio. Data show the mean +/- SD of three independent experiments, ns: non-significant p>0.05.
(PDF)

S9 Fig Roles of ALPK1 in different pathways activating NF-κB. **A**) ALPK1 depletion has no effect on PMA-induced NF-κB activation. Cells were transfected with control or ALPK1 siRNAs, stimulated with PMA (100 ng/ml) for 1 hour and stained for NF-κB p65. Data show the mean +/- SD of three independent experiments, ns: non-significant p>0.05. **B**) ALPK1 depletion has no effect on TNFα-induced NF-κB activation. Cells were treated as in A and stimulated with indicated concentrations of TNFα for 30 min. Data show the mean +/- SD of three independent experiments, ns: non-significant p>0.05. **C**) ALPK1 depletion has a moderate inhibitory effect on C12-iE-DAP-induced NF-κB activation. Cells were treated as in A and stimulated with indicated concentrations of C12-iE-DAP for 1 hour. Data show the mean +/- SD of three independent experiments, ns: non-significant p>0.05, p*<0.05.
(PDF)

S10 Fig TIFA oligomers are not co-localized with Lysosomal-associated membrane protein 1 (LAMP-1). HeLa cells were left uninfected or infected for 1 hour with *S*. *flexneri* expressing dsRed at MOI 0.5. Cells were stained for TIFA and LAMP1. Scale bar, 10 μm.
(PDF)

S1 Table Results of the genome wide RNAi screen. Z-scored values of total cell counts, infection rates and IL-8 measurements obtained with CellProfiler for all genes targeted by the siRNA library (see Materials and Methods). Data correspond to the mean of duplicate screening data. TIFA, TRAF6 and ALPK1 are shown in red. The positive controls RelA (NF-κB p65) and MAP3K7 (TAK1) are shown in blue.
(XLSX)

S2 Table Results of the human kinome screen. Data show Z-scored values of total cell counts, infection rates and IL-8 measurements obtained with CellProfiler for all genes targeted by the human kinome library (see Materials and Methods). Data are shown for all 3 individual sequences/gene or pooled.
(XLSX)

S3 Table Primers used in this study. (PDF)
