# Supplementary material for: ALPK1 controls TIFA/TRAF6-dependent innate immunity against heptose-1,7-bisphosphate of gram-negative bacteria
Source: PLoS Pathog. 2017 Feb 21;13(2):e1006224. doi: 10.1371/journal.ppat.1006224 (PMC5336308; doi:10.1371/journal.ppat.1006224)
Supplement: S1 Fig — HeLa cells were infected for 3.5 hours with S. flexneri ΔvirG expressing dsRed under the control of the uhpT promoter (in green). After fixation, cells were stained for F-actin (in grey), DNA (in blue) and IL-8 (in red). Scale bars, 20 μm. (PDF) [file ppat.1006224.s001.pdf]

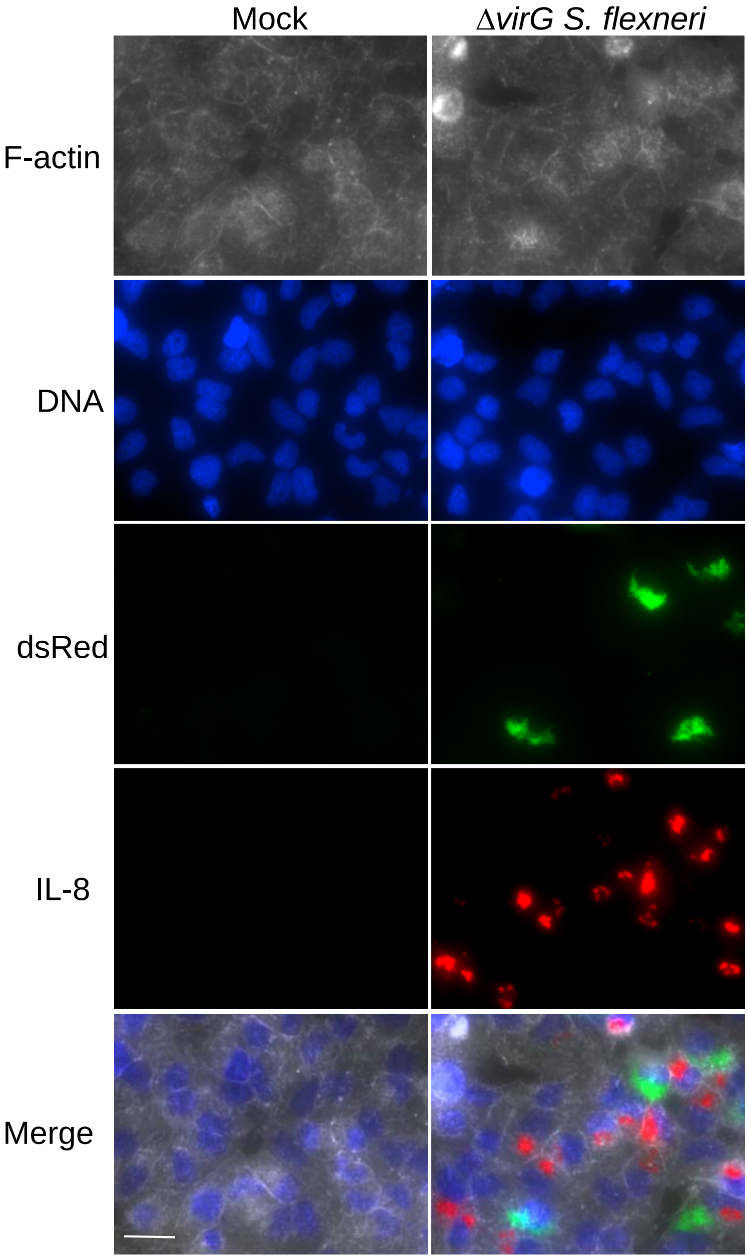

Figure S1: Images illustrating the assay of the RNAi screen.
HeLa cells were infected for 3.5 hours with *S. flexneri* Δ*virG* expressing dsRed under the control of the *uhpT* promoter (in green). After fixation, cells were stained for F-actin (in grey), DNA (in blue) and IL-8 (in red). Scale bars, 20 μm.
