# Supplementary material for: ALPK1 controls TIFA/TRAF6-dependent innate immunity against heptose-1,7-bisphosphate of gram-negative bacteria
Source: PLoS Pathog. 2017 Feb 21;13(2):e1006224. doi: 10.1371/journal.ppat.1006224 (PMC5336308; doi:10.1371/journal.ppat.1006224)
Supplement: S2 Fig — HeLa cells were transfected for 72 hours with control, TIFA- or ALPK1-targeting siRNAs. HeLa cells were infected for 3.5 hours with ΔvirG S. flexneri expressing dsRed. After fixation, cells were stained for F-actin and DNA. Infection rate was evaluated by automated image analysis. Data correspond to the mean +/- SD of 3 independent experiments, NS = non-significant p>0.05. (PDF) [file ppat.1006224.s002.pdf]

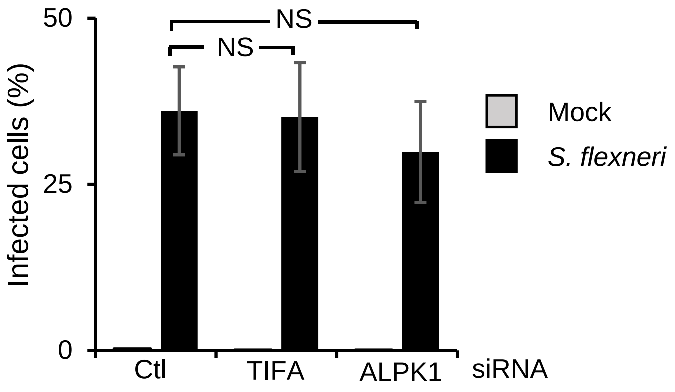

Figure S2: Silencing TIFA or ALPK1 has no significant effect on *S. flexneri* entry
HeLa cells were transfected for 72 hours with control, TIFA or ALPK1-targeting siRNAs. HeLa cells were infected for 3.5 hours with Δ*virG S. flexneri* expressing dsRed. After fixation, cells were stained for F-actin and DNA. Infection rate was evaluated by automated image analysis. Data correspond to the mean +/- SD of 3 independent experiments, NS = non-significant p>0.05.
