# Supplementary material for: ALPK1 controls TIFA/TRAF6-dependent innate immunity against heptose-1,7-bisphosphate of gram-negative bacteria
Source: PLoS Pathog. 2017 Feb 21;13(2):e1006224. doi: 10.1371/journal.ppat.1006224 (PMC5336308; doi:10.1371/journal.ppat.1006224)
Supplement: S3 Fig — Caco-2 cells were transfected with a wild-type TIFA cDNA construct and infected for 3 hours with ΔvirG S. flexneri expressing dsRed (in blue). After fixation, cells were co-stained for TIFA (in green) and NF-κB p65 (in red). Scale bars, 20 μm. (PDF) [file ppat.1006224.s003.pdf]

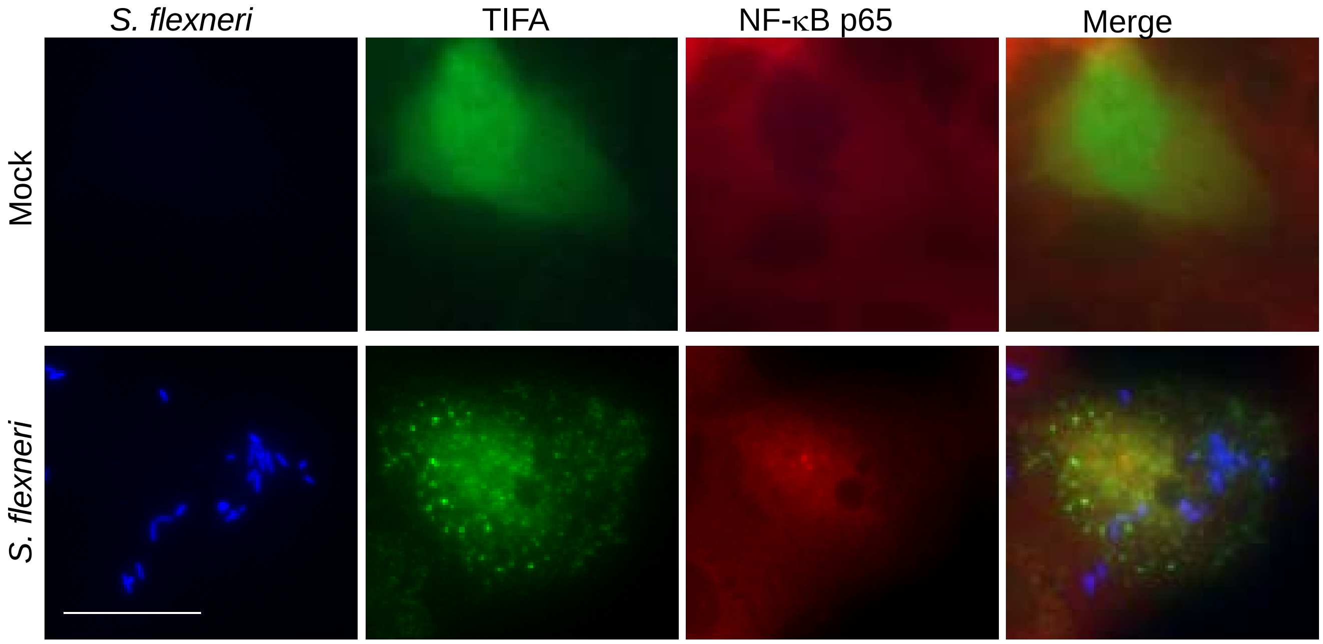

Figure S3: TIFA oligomerization after *S. flexneri* infection of Caco-2 cells.
Caco-2 cells were transfected with a wild-type TIFA cDNA construct and infected for 3 hours with Δ*virG* *S. flexneri* expressing dsRed (in blue). After fixation, they were co-stained for TIFA (in green) and NF-κB p65 (in red). Scale bars, 20 μm.
