# Supplementary material for: ALPK1 controls TIFA/TRAF6-dependent innate immunity against heptose-1,7-bisphosphate of gram-negative bacteria
Source: PLoS Pathog. 2017 Feb 21;13(2):e1006224. doi: 10.1371/journal.ppat.1006224 (PMC5336308; doi:10.1371/journal.ppat.1006224)
Supplement: S4 Fig — A) ADP-Heptose biosynthetic pathway. B) S. typhimurium-induced IL-8 production is largely HBP-dependent. HeLa cells were infected with S. typhimurium for 4 hours, fixed and stained for intracellular IL-8. Data correspond to the mean +/- SD of three independent experiments, p**<0.005. C) Infectivity of wt, ΔhldE and ΔwaaC S. flexneri. HeLa cells were infected with wt (MOI 100), ΔhldE (MOI 1) and ΔwaaC (MOI 1) S. flexneri expressing dsRed. The rate of infection was quantified automatically. Data correspond to the mean +/- SD of three independent experiments, non-significant p>0.05. D) S. flexneri-induced IL-8 production is largely HBP-dependent. HeLa cells were infected with S. flexneri for 4 hours, fixed and stained for intracellular IL-8. Data correspond to the mean +/- SD of three independent experiments, p**<0.005. (PDF) [file ppat.1006224.s004.pdf]

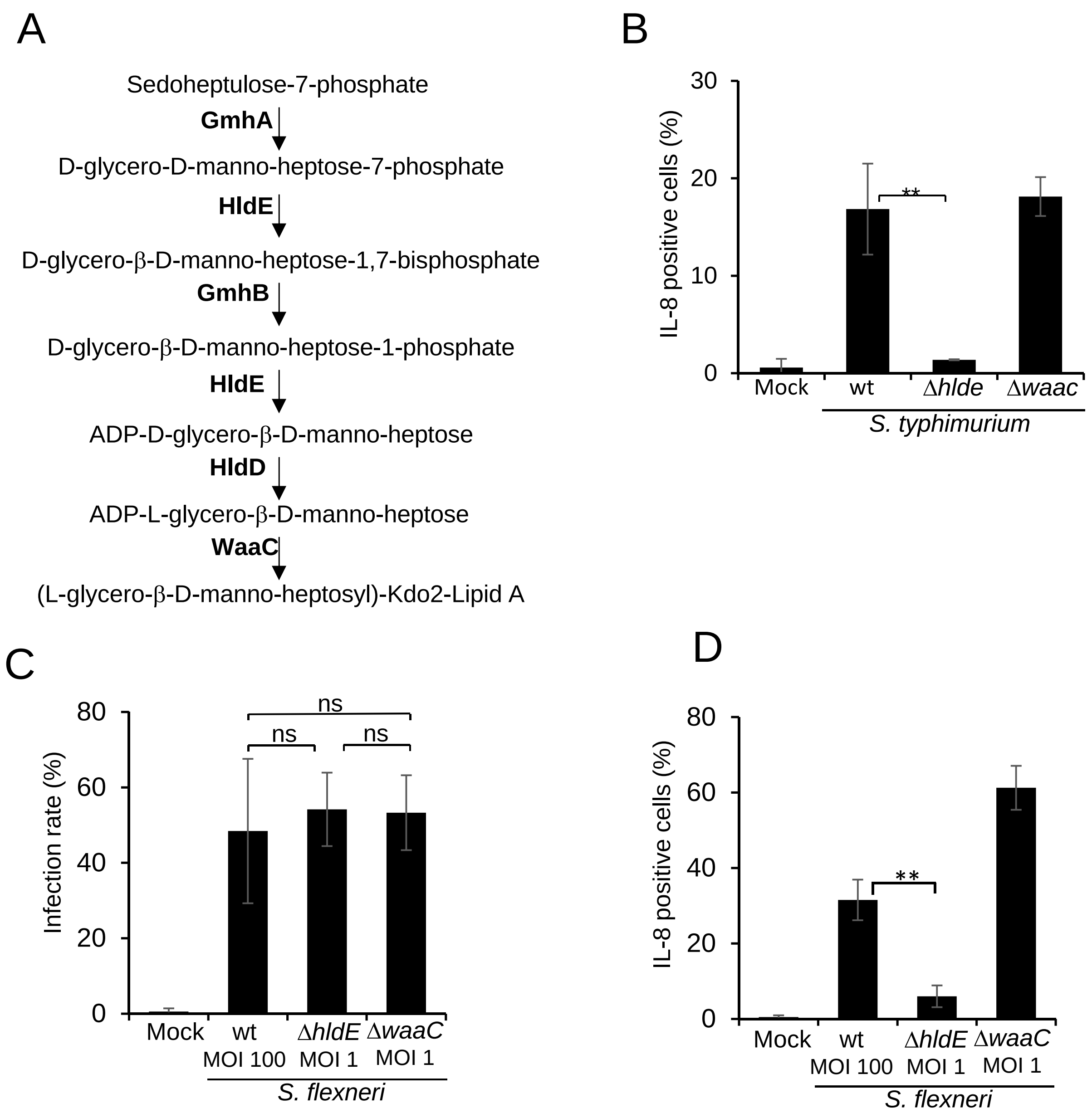

Figure S4: *S. typhimurium* and *S. flexneri*-induced IL-8 production is largely HBP-dependent.
A) ADP-Heptose biosynthetic pathway. **B**) *S. typhimurium*-induced IL-8 production is largely HBP-dependent. HeLa cells were infected with *S. typhimurium* for 4 hours, fixed and stained for intracellular IL-8. Data correspond to the mean +/- SD of three independent experiments, p**<0.005. **C**) Infectivity of wt, Δ*hldE* and Δ*waaC* *S. flexneri*. HeLa cells were infected with wt (MOI 100), Δ*hldE* (MOI 1) and Δ*waaC* (MOI 1) *S. flexneri* expressing dsRed. The rate of infection was quantified automatically. Data correspond to the mean +/- SD of three independent experiments, non-significant p>0.05. **D**) *S. flexneri*-induced IL-8 production is largely HBP-dependent. HeLa cells were infected with *S. flexneri* for 4 hours, fixed and stained for intracellular IL-8. Data correspond to the mean +/- SD of three independent experiments, p**<0.005.
