# Supplementary material for: ALPK1 controls TIFA/TRAF6-dependent innate immunity against heptose-1,7-bisphosphate of gram-negative bacteria
Source: PLoS Pathog. 2017 Feb 21;13(2):e1006224. doi: 10.1371/journal.ppat.1006224 (PMC5336308; doi:10.1371/journal.ppat.1006224)
Supplement: S5 Fig — A) HeLa cells were transfected with a wild-type TIFA cDNA construct. After 24 hours, they were infected with wild-type, ΔhldE or ΔgmhB S. typhimurium expressing dsRed under the control of the uhpT promoter. The fraction of infected cells showing TIFA punctuates was manually evaluated. Data correspond to the mean +/- SD of 3 independent experiments. B) HeLa cells were transfected with a wild-type TIFA cDNA construct. After 24 hours, they were infected with wild-type, ΔhldE or ΔgmhB S. flexneri expressing dsRed. The fraction of infected cells showing TIFA punctuates was manually evaluated. Data correspond to the mean +/- SD 3 independent experiments. (PDF) [file ppat.1006224.s005.pdf]

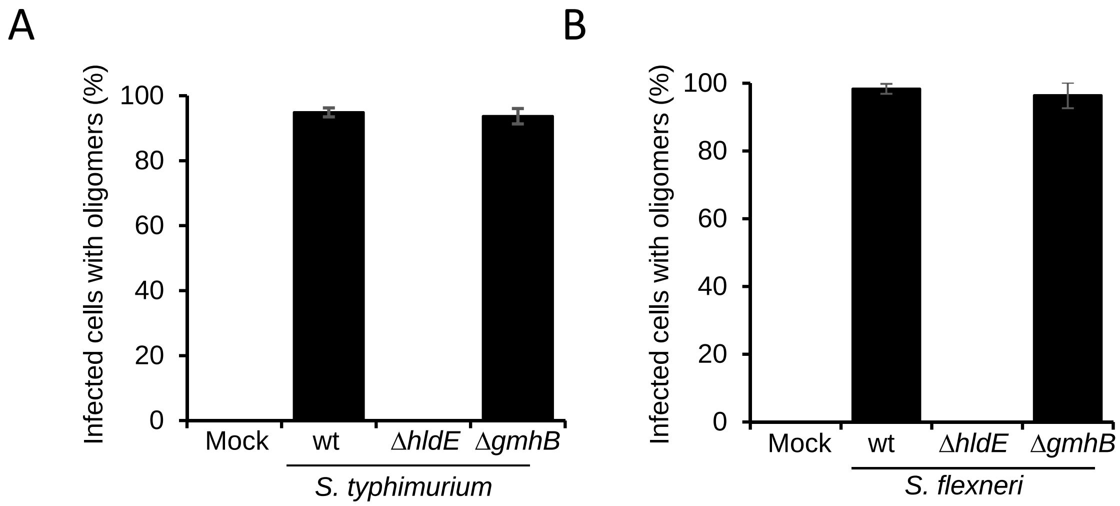

Figure S5: TIFA oligomerization is HBP-dependent.
A) HeLa cells were transfected with a wild-type TIFA cDNA construct. After 24 hours, they were infected with wild-type, Δ*hldE* or Δ*gmhB S. typhimurium* expressing dsRed under the control of the *uhpT* promoter. The fraction of infected cells showing TIFA punctuates was manually evaluated. Data correspond to the mean +/- SD of 3 independent experiments.
B) HeLa cells were transfected with a wild-type TIFA cDNA construct. After 24 hours, they were infected with wild-type, Δ*hldE* or Δ*gmhB S. flexneri* expressing dsRed. The fraction of infected cells showing TIFA punctuates was manually evaluated. Data correspond to the mean +/- SD 3 independent experiments.
