# Supplementary material for: ALPK1 controls TIFA/TRAF6-dependent innate immunity against heptose-1,7-bisphosphate of gram-negative bacteria
Source: PLoS Pathog. 2017 Feb 21;13(2):e1006224. doi: 10.1371/journal.ppat.1006224 (PMC5336308; doi:10.1371/journal.ppat.1006224)
Supplement: S6 Fig — A) HeLa cells were infected or not with S. flexneri for 6 hours with wt (MOI 10), ΔhldE (MOI 0.1) and ΔwaaC (MOI 0.1) S. flexneri. Cytokine secretion was measured in the supernatant of infected cells by a multiplex cytokine assay. Data correspond to the mean +/- SD of triplicates, p**<0.005, p***<0.0005. # indicates not detected. B) Caco-2 cells were infected or not with S. flexneri for 6 hours with wt (MOI 10), ΔhldE (MOI 0.1) and ΔwaaC (MOI 0.1) S. flexneri. Cytokine secretion was measured in the supernatant of infected cells by a multiplex cytokine assay. Data correspond to the mean +/- SD of triplicates, p**<0.005. IL-1β, IFNγ and IL-6 were not detected in Caco-2 cells. (PDF) [file ppat.1006224.s006.pdf]

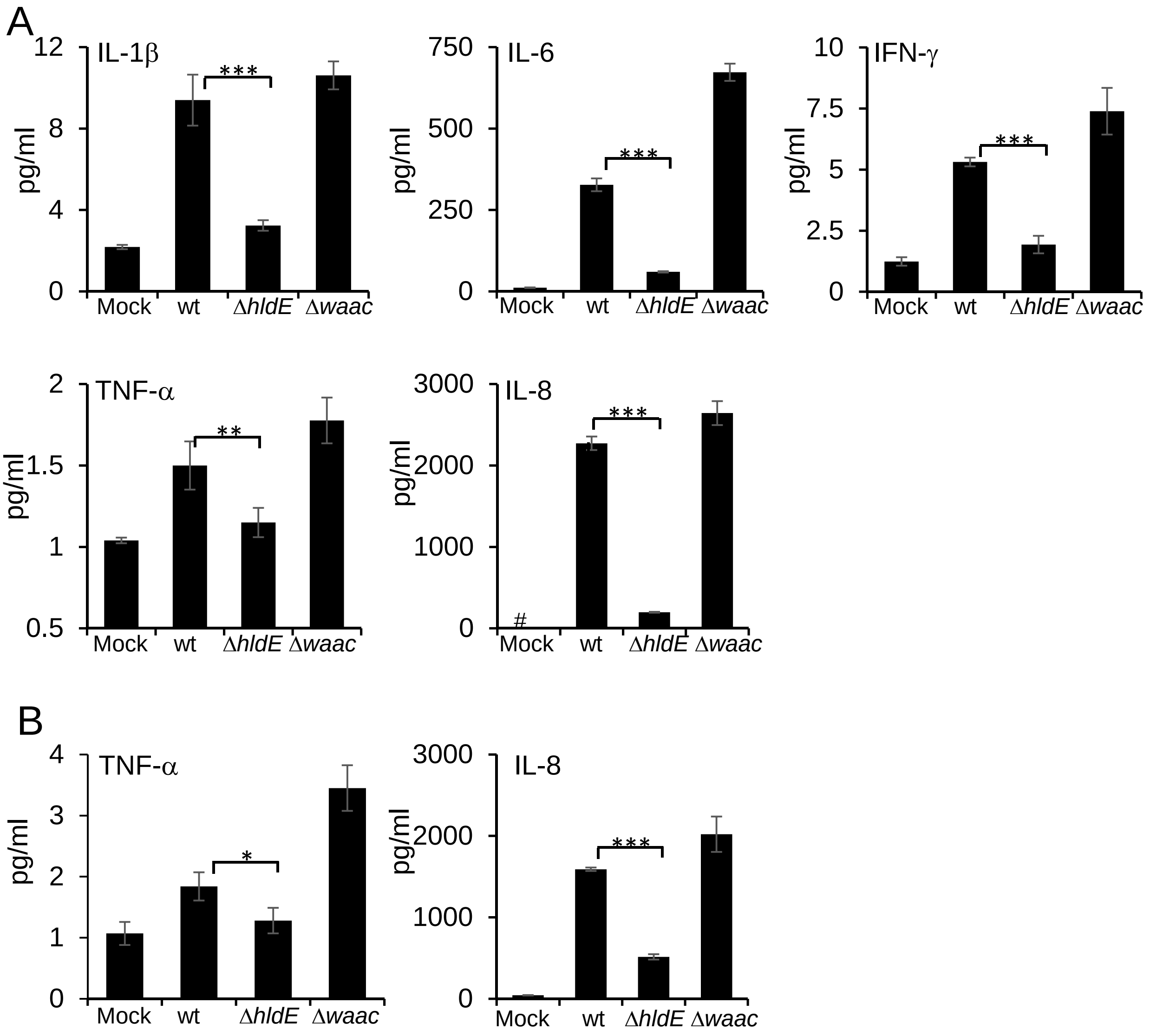

Figure S6: The production of cytokines induced by *S. flexneri* infection is largely HBP-dependent.

A) HeLa cells were infected or not with *S. flexneri* for 6 hours with wt (MOI 10), Δ*hldE* (MOI 0.1) and Δ*waaC* (MOI 0.1) *S. flexneri.* Cytokine secretion was measured in the supernatant of infected cells by a multiplex cytokine assay. Data correspond to the mean +/- SD of triplicates, p**<0.005, p***<0.0005. # indicates not detected. **B**) Caco-2 cells were infected or not with *S. flexneri* for 6 hours with wt (MOI 10), Δ*hldE* (MOI 0.1) and Δ*waaC* (MOI 0.1) *S. flexneri*. Cytokine secretion was measured in the supernatant of infected cells by a multiplex cytokine assay. Data correspond to the mean +/- SD of triplicates, p**<0.005. IL-1β, IFNγ and IL-6 were not detected in Caco-2 cells.
