# Supplementary material for: ALPK1 controls TIFA/TRAF6-dependent innate immunity against heptose-1,7-bisphosphate of gram-negative bacteria
Source: PLoS Pathog. 2017 Feb 21;13(2):e1006224. doi: 10.1371/journal.ppat.1006224 (PMC5336308; doi:10.1371/journal.ppat.1006224)
Supplement: S7 Fig — A) ELISA assay showing that S. flexneri-induced IL-8 expression is ALPK1-dependent. HeLa cells were transfected with control or ALPK1 siRNA, and infected, or not, with S. flexneri for 6 hours. IL-8 secretion was measured in the supernatant of infected cells by ELISA. Data correspond to the mean +/- SD of three independent experiments, p*<0.05. B) HeLa cells were transfected with control or ALPK1 siRNA, and infected or not with S. flexneri for 6 hours. Cytokine secretion was measured in the supernatant of infected cells by a multiplex cytokine assay. Data correspond to the mean +/- SD of triplicates, p**<0.005, p***<0.0005. # indicates not detected. (PDF) [file ppat.1006224.s007.pdf]

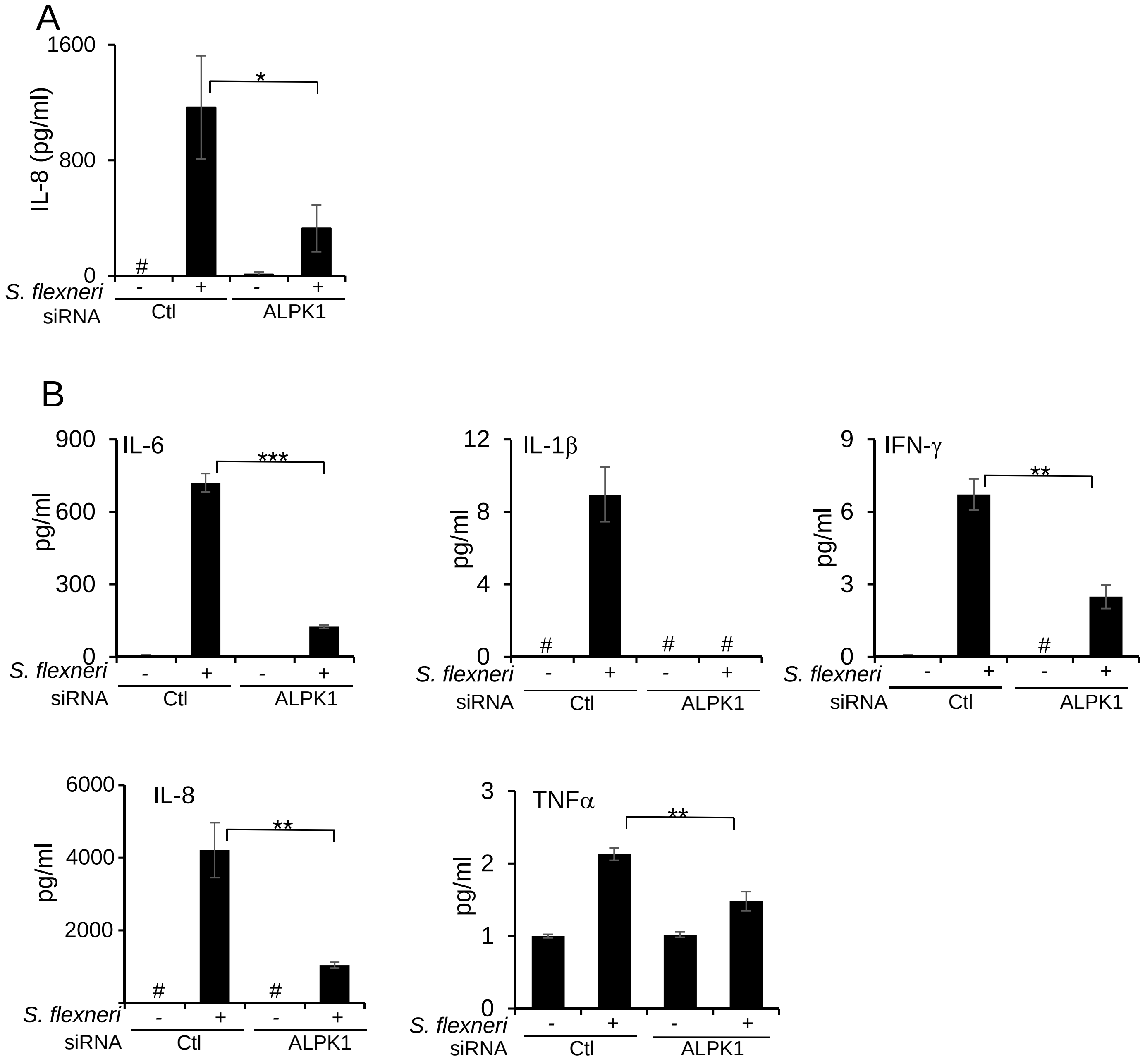

Figure S7: The production of cytokines induced by *S. flexneri* infection depends on ALPK1.
A) ELISA assay showing that *S. flexneri*-induced IL-8 expression is ALPK1-dependent. HeLa cells were transfected with control or ALPK1 siRNA, and infected or not with *S. flexneri* for 6 hours. IL-8 secretion was measured in the supernatant of infected cells by ELISA. Data correspond to the mean +/- SD of three independent experiments, p*<0.05. **B**) HeLa cells were transfected with control or ALPK1 siRNA, and infected or not with *S. flexneri* for 6 hours. Cytokine secretion was measured in the supernatant of infected cells by a multiplex cytokine assay. Data correspond to the mean +/- SD of triplicates, p**<0.005, p***<0.0005. # indicates not detected.
