# Supplementary material for: ALPK1 controls TIFA/TRAF6-dependent innate immunity against heptose-1,7-bisphosphate of gram-negative bacteria
Source: PLoS Pathog. 2017 Feb 21;13(2):e1006224. doi: 10.1371/journal.ppat.1006224 (PMC5336308; doi:10.1371/journal.ppat.1006224)
Supplement: S8 Fig — A) ALPK1 is not involved in L. monocytogenes-induced IL-8 expression. HeLa cells were transfected for 72 hours with control or ALPK1-targeting siRNAs and infected for 3.5 hours with L. monocytogenes expressing GFP. After fixation, cells were stained for F-actin, DNA, and IL-8. IL-8 was quantified by automated image analysis. Data correspond to the mean +/- SD of triplicate wells and the graph is representative of 3 independent experiments, ns: non-significant p>0.05. B) ALPK1 is not involved in L. monocytogenes-induced NF-κB activation. Cells were treated as in A but stained for NF-κB p65 after one hour of infection. Quantification of the NF-κΒ p65 nuclear/cytoplasmic fluorescence intensity ratio. Data show the mean +/- SD of three independent experiments, ns: non-significant p>0.05. (PDF) [file ppat.1006224.s008.pdf]

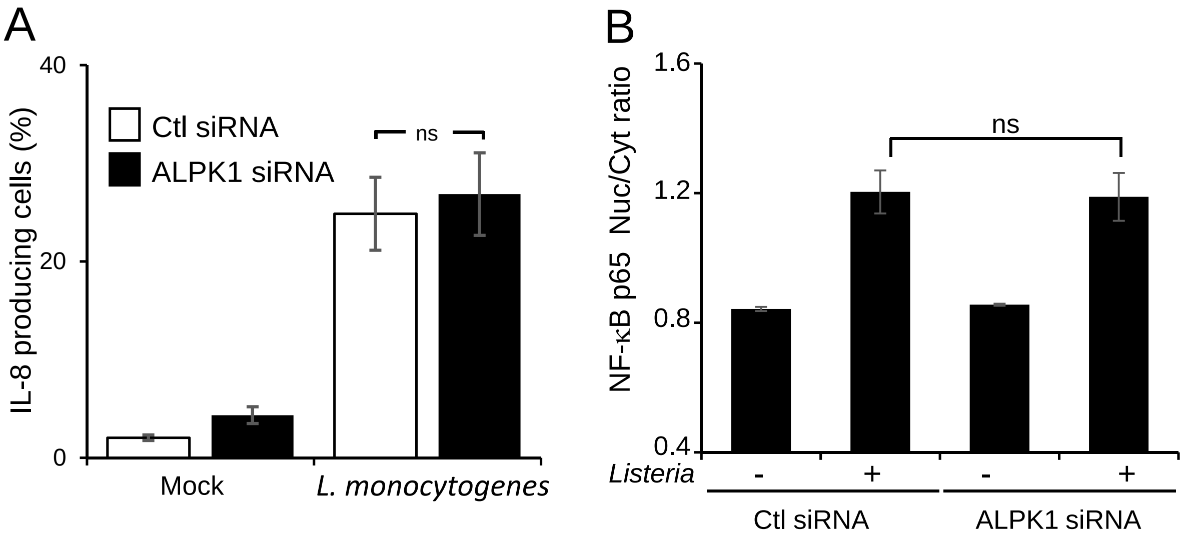

Figure S8: ALPK1 is not involved in *L. monocytogenes*-induced inflammation

A) ALPK1 is not involved in *L. monocytogenes*-induced IL-8 expression. HeLa cells were transfected for 72 hours with control or ALPK1-targeting siRNAs and infected for 3.5 hours with *L. monocytogenes* expressing GFP. After fixation, cells were stained for F-actin, DNA, and IL-8. IL-8 was quantified by automated image analysis. Data correspond to the mean +/- SD of triplicate wells, graph representative of 3 independent experiments, ns: non-significant p>0.05. **B**) ALPK1 is not involved in *L. monocytogenes*-induced NF-κB activation. Cells were treated as in A but stained for NF-κB p65 after one hour of infection. Quantification of the NF-κB p65 nuclear/cytoplasmic fluorescence intensity ratio. Data show the mean +/- SD of three independent experiments, ns: non-significant p>0.05.
