# Supplementary material for: ALPK1 controls TIFA/TRAF6-dependent innate immunity against heptose-1,7-bisphosphate of gram-negative bacteria
Source: PLoS Pathog. 2017 Feb 21;13(2):e1006224. doi: 10.1371/journal.ppat.1006224 (PMC5336308; doi:10.1371/journal.ppat.1006224)
Supplement: S9 Fig — A) ALPK1 depletion has no effect on PMA-induced NF-κB activation. Cells were transfected with control or ALPK1 siRNAs, stimulated with PMA (100 ng/ml) for 1 hour and stained for NF-κB p65. Data show the mean +/- SD of three independent experiments, ns: non-significant p>0.05. B) ALPK1 depletion has no effect on TNFα-induced NF-κB activation. Cells were treated as in A and stimulated with indicated concentrations of TNFα for 30 min. Data show the mean +/- SD of three independent experiments, ns: non-significant p>0.05. C) ALPK1 depletion has a moderate inhibitory effect on C12-iE-DAP-induced NF-κB activation. Cells were treated as in A and stimulated with indicated concentrations of C12-iE-DAP for 1 hour. Data show the mean +/- SD of three independent experiments, ns: non-significant p>0.05, p*<0.05. (PDF) [file ppat.1006224.s009.pdf]

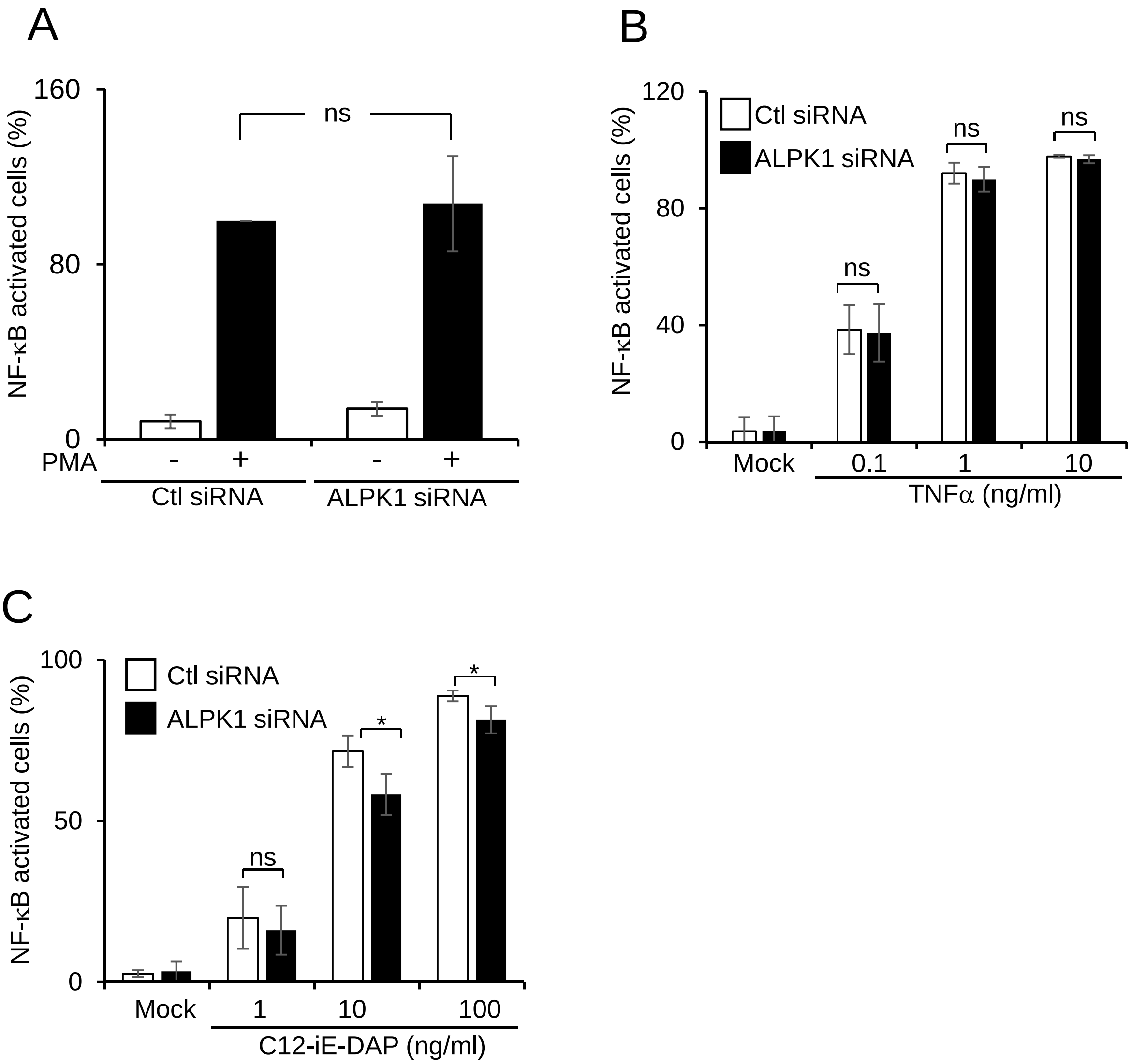

Figure S9: Roles of ALPK1 in different pathways activating NF-κB.

A) ALPK1 depletion has no effect on PMA-induced NF-κB activation. Cells were transfected with control or ALPK1 siRNAs, stimulated with PMA (100 ng/ml) for 1 hour and stained for NF-κB p65. Data show the mean +/- SD of three independent experiments, ns: non-significant p>0.05. **B**) ALPK1 depletion has no effect on TNFα-induced NF-κB activation. Cells were treated as in A and stimulated with indicated concentrations of TNFα for 30 min. Data show the mean +/- SD of three independent experiments, ns: non-significant p>0.05. **C**) ALPK1 depletion has a moderate inhibitory effect on C12-iE-DAP-induced NF-κB activation. Cells were treated as in A and stimulated with indicated concentrations of C12-iE-DAP for 1 hour. Data show the mean +/- SD of three independent experiments, ns: non-significant p>0.05, p*<0.05.
