# Supplementary material for: ALPK1 controls TIFA/TRAF6-dependent innate immunity against heptose-1,7-bisphosphate of gram-negative bacteria
Source: PLoS Pathog. 2017 Feb 21;13(2):e1006224. doi: 10.1371/journal.ppat.1006224 (PMC5336308; doi:10.1371/journal.ppat.1006224)
Supplement: S10 Fig — HeLa cells were left uninfected or infected for 1 hour with S. flexneri expressing dsRed at MOI 0.5. Cells were stained for TIFA and LAMP1. Scale bar, 10 μm. (PDF) [file ppat.1006224.s010.pdf]

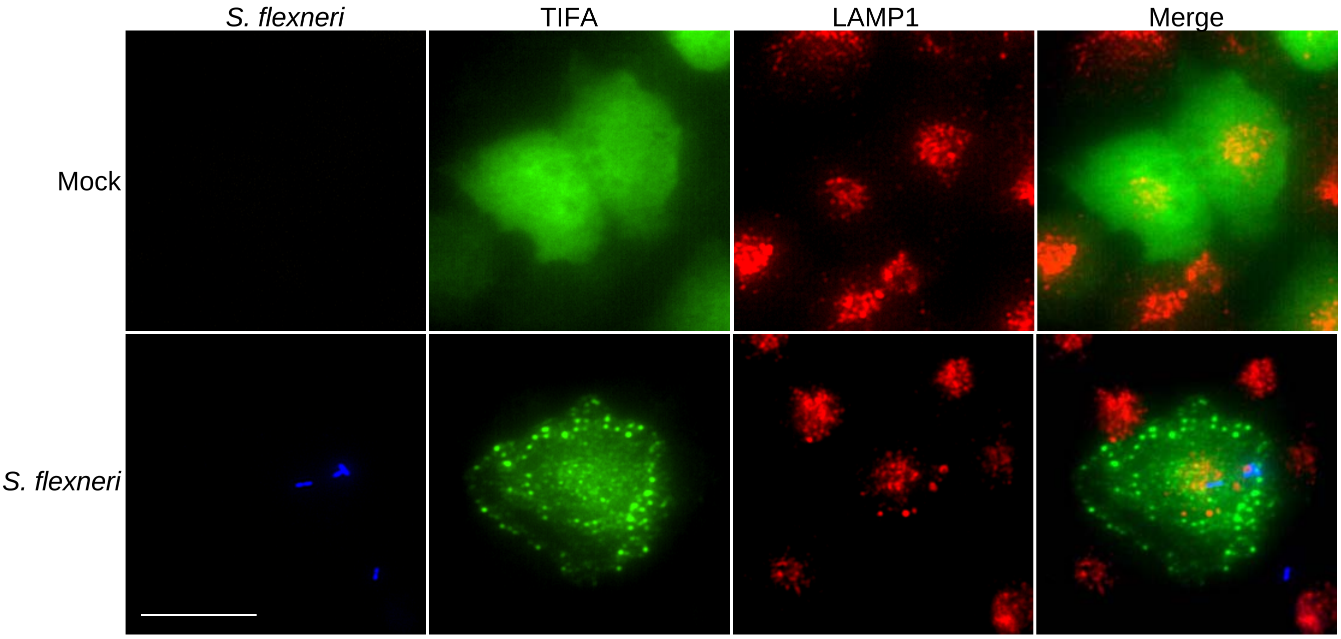

Figure S10: TIFA oligomers are not co-localized with *Lysosomal*-associated membrane protein 1 (*LAMP-1*). HeLa cells were left uninfected or infected for 1 hour with *S. flexneri* expressing dsRed at MOI 0.5. Cells were stained for TIFA and LAMP1. Bar, 10 μm.
