# Supplementary material for: ALPK1 controls TIFA/TRAF6-dependent innate immunity against heptose-1,7-bisphosphate of gram-negative bacteria
Source: PLoS Pathog. 2017 Feb 21;13(2):e1006224. doi: 10.1371/journal.ppat.1006224 (PMC5336308; doi:10.1371/journal.ppat.1006224)
Supplement: S3 Table — (PDF) [file ppat.1006224.s013.pdf]

Table S3: Table listing the primers used in this study.

Primer	Sequence
S. flexneri Δ*hldE* Forward	ATGGTATTATCGCGCGCAAATTTTGAATCTCTCAGGAGACAGGAATGAAATGTGTAGGCTGGAGCTGCTTCG
S. flexneri Δ*hldE* Reverse	TAGGCCTGCCTGCTACGAAGCGAGATCTGTGAACCGCTTTCCGGTTAGCCCATATGAATATCCTCCTTAG
S. flexneri Δ*gmhB* Forward	AGGCATCCAGACGTCTAAATCAATCAGGTTTATGCGAAGAGCACTTTCTTGCAGGTCGAAACATGCGATACTAGCGTCCC<br>ATGCCTTATTAAGGAGCTATAAAAGGTGGCGAAGAGCTGTGTAGGCTGGAGCTGCTTCG
S. flexneri Δ*gmhB* Reverse	GTGTGAAGTGATTCACATCCGCCGTGTCGATGGAGGCGCATTATAGGGAGTCGGCTCAGGAAGACAAGCGGAAAAATGCA<br>TTTTTATTTCAACCGTTCATCTTTTAATCATTGCGCCGGCATATGAATATCCTCCTTAG
S. flexneri *∆waaC Forward*	AAAGGTGACGCTGCGGAGGGTTATCACCAGAGCTTGATCGACATTACTCCCCAGCGCGTACTGGAAGAACTCAACGCGCTATTGTTACAAGAGGAAGCCTGACGGATGCGGGTTTTGTGTGTAGGCTGGAGCTGCTTCG
S. flexneri *∆waaC Reverse*	CCTTGTAATCCCACCCAAAAAATAGGTGGTAATGAATAAGAATACGAGAGCTCTGTTCCAGTACGCTATCCAGTTTTTTTTCTCGAGAGAGAAAAATAATGTTGAGGTCATCTTATCTCCATATGAATATCCTCCTTAG
N. meningitidis hldA Forward overexpression	CCCAAGCTTAATGTCCGCCAAGTTCCAAC
N. meningitidis hldA Reverse overexpression	CCGGAATTCCTACATTGTTGATTGCCCTGA
S. typhimurim Δ*hldE* Forward	GCTATTATCGCGCGCAAATTTTGAATCTCTCAGGAGACAGGAATGAAATGTGTAGGCTGGAGCTGCTTCG
S. typhimurim Δ*hldE* Reverse	CAAAATCAGCGCCCGCCGCGTCTGTAACGCCTGATGACGCGGTTTTACTTCATATGAATATCCTCCTTAG
S. typhimurim Δ*gmhB* Forward	TGCGATACTAGCGCCACTTGCTTTACTCAGGAGCTATAAAAGGTGGCATGTGTAGGCTGGAGCTGCTTCG
S. typhimurim Δ*gmhB* Reverse	AGAATGCATTTGTGTTTTAGCCGCTCATCTTTTATCCATAACGCTTATTTCATATGAATATCCTCCTTAG
S. typhimurim Δ*waaC* Forward	CAAAGTGCGTAAAGGTGATACGGCGCAAGGCTATCACCAGAGCCTGATCGATATCACGCCGCAGCGGGTTCTGGAAGAGCTTCATTCGCTGTTGTCGGAAGAGGGCGTTTAATGCGGTGTGTAGGCTGGAGCTGCTTCG
S. typhimurim Δ*waaC* Reverse	AAAAGAAAAACCAGCGCTTTATTCCAGATCGGCTTCCATTTCTCTTTATTTAACGTTAATGATGTGGTTAGCATCTTTTCTCCACAATAGGTTTGGGATGAGACAGAGTCTCTTTAATGCATATGAATATCCTCCTTAG
TIFA_BamHI_F	GCGGGATCCCGATGACCAGTTTTGAAGATGCTGA
TIFA_R2	GGGAGTTTCTCTCGATTGAAACTT

TIFA_F2	TTTCAGTCAATAAGTTTCAATCGAGAGA
TIFA_XbaI_R	GCTCTAGATCATGACTCATTTTCATCCATTTCTGT
TIFA_EA_XbaI_R	GCTCTAGATCATGACTCATTTTCATCCATTGCT
ALPK1 YFP FL siRNA resistant 5 mutations (t761a_c762g_c763t_a767t_g768c) Forward	5'-ggattgtttttaaacttttcataatcgttcttggacatactaacaaagatgtctgccagtatacctagcgacg-3'
ALPK1 YFP FL siRNA resistant 5 mutations (t761a_c762g_c763t_a767t_g768c) Reverse	5'-cgtcgctaggtatactggcagacatctttgttagtatgtccaagaacgattatgaaaagtttaaaaacaatcc-3'
ALPK1 YFP Delta Kinase : t3059g Forward	5'-agttcagatttttttgaatattttcacaaaagagcactatgtgctcgg-3'
ALPK1 YFP Delta Kinase : t3059g Reverse	5'-ccgagcacatagtgctcttttgtgaaaatattcaaaaaaatctgaact-3'
